# Optimizing green hydrogen production: a comparative analysis of MPPT control strategies for PV-powered PEM electrolyzers using differentiated creative search optimization algorithm

**DOI:** 10.1038/s41598-026-46999-5

**Published:** 2026-05-14

**Authors:** Alaa Abdelhamid Mohamed, Mohammed Hamouda Ali, Ahmed I. Omar, Mohammed Mehanna

**Affiliations:** 1 Electrical Power and Machines Engineering Department, Higher Institute of Engineering, EL-Shorouk Academy, Cairo, Egypt; 2 Electrical Engineering Department, Faculty of Engineering, Al-Azhar University, Cairo, Egypt

**Keywords:** Green hydrogen production, PEM electrolyzer, Photovoltaic system, Maximum power point tracking (MPPT), Fuzzy logic controller (FLC), Metaheuristic optimization, Energy science and technology, Engineering

## Abstract

Hydrogen is one of the potential clean energy sources that might help to address two critical global issues: energy scarcity and environmental concerns. Using fossil fuels for hydrogen generation has drawbacks, such as increased greenhouse gas emissions throughout the process. As a result, finding clean, sustainable, and dependable hydrogen generation technology cheaply and with zero emissions has become critical. The purpose of this study is to analyze hydrogen generation from solar energy. Mainly focus on PEM electrolyzer as a source of hydrogen and solar energy as a source of power fed to electrolyzer, so it is necessary to ensure that PV operate at maximum power or close to it, so we used P&O MPPT technique with several controllers like fuzzy logic (FL), proportional integer (PI) and fraction order proportional integer (FOPI) controllers. To achieve optimal tuning for the final two controller parameters, differentiated creative search optimization algorithm (DCSO) is applied and compared to other algorithms such as PSO and GWO. When comparing the outcomes, it was revealed that PI-DCSO is the best, with 6987 W produced power, followed by FOPI-DCSO with 6767 W, and the FLC with 6296 W output power, as detailed in the result chapter, which also contains a comparison of PV production under varying conditions, and a comparison of PEM electrolyzer under different conditions.

## Introduction

**Fig. 1:**
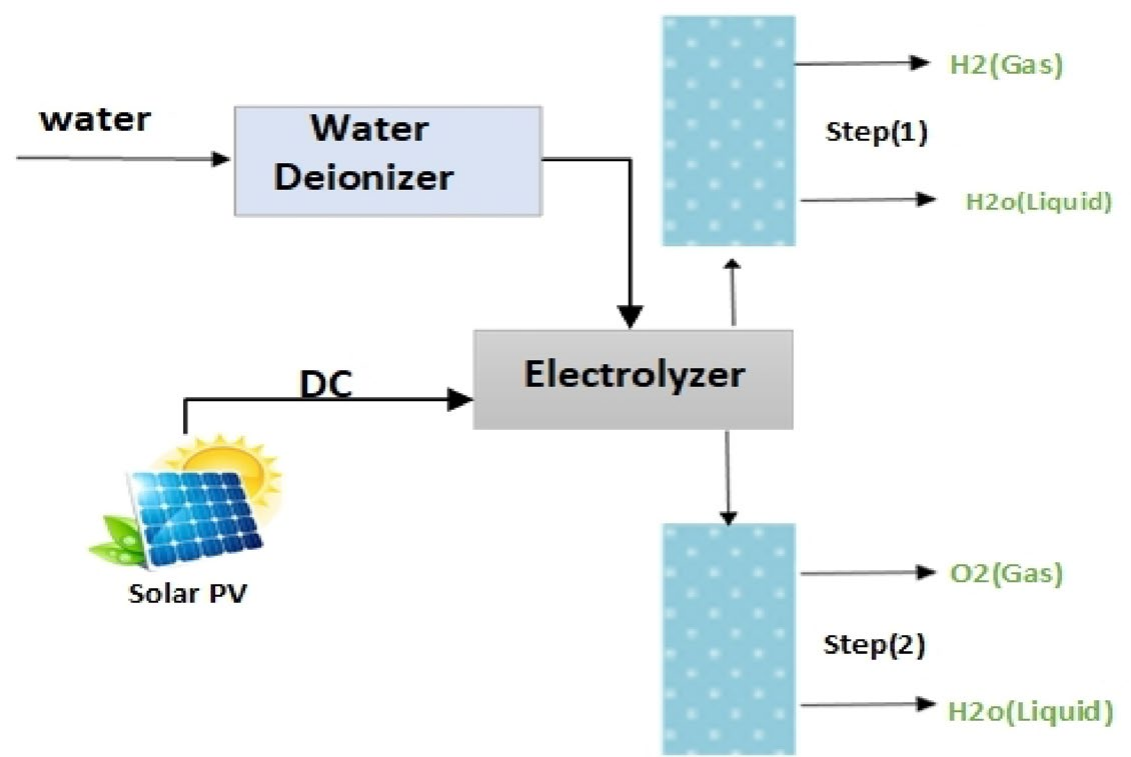
Schematic diagram of the hydrogen production process.

The entire world is looking to renewable energy in the present day to substitute fossil fuels with green energy instead of conventional forms of energy. Given the limited supply of fossil fuels, rising fuel prices, and environmental issues brought on by the world’s energy use, hydrogen is one of the most promising energy sources for creating a carbon-free energy system^1,2^. Proton exchange membrane (PEM) and Alkaline (AL) water electrolyzers are low-temperature electrochemical methods for splitting water into hydrogen and oxygen^3^. Solar photovoltaic (PV) power represents one of the cheapest and most widely deployed sources of renewable electricity, with over 520 GW of cumulative installed capacity worldwide. Therefore, it is considered the prime energy vector to power green hydrogen production. One of the downsides of solar energy is the difficulty in moving it from one region to another, as it is dependent on location and, more importantly, weather. Unlike hydrogen electrolyzers, in this study, solar energy is employed to provide sustained power to feed the electrolyzers, allowing the entire system to be powered by clean energy. Stabilizing and guaranteeing a steady energy supply is feasible by employing solar energy to electrolyze hydrogen. Water electrolysis powered by sunlight can replace the electrical requirements of traditional electricity sources while also increasing the overall effectiveness of energy^4^. electrolysis utilizing solar power. Maximum Fig. 1. Simulink model for hydrogen production to generate all possible power they are capable of, such as MPPT^5^.AS solar energy is Power Point Tracking (MPPT) is a control system-based approach that allows PV modules critical for hydrogen production, it was utilized in Perturb and Observation (P&O) MPPT and controlled it in this study. Control (P&O MPPT) uses fractional order proportional integer controllers (FOPI) and proportional integer controllers (PI), with tuning their parameters using various algorithms such as “DCSO, PSO, GWO “, and comparing their results. The last control (P&O MPPT) employed a fuzzy logic controller (FLC) and compared its output with the preceding results.

## Literature review

The literature describes hydrogen production processes, such as electrolysis, solar DC/DC Converter Varieties, and MPPT control strategies. The most common electrolyzers are AL and PEM electrolyzers^6,7^. AL is an established technology with lower capital costs; however, it is less suited to handling dynamic operations due to its slower response time and narrower load range^8^. Cost forecast for low-temperature electrolysis using technology-driven bottom-up programming for PEM and alkaline water electrolysis systems^9^. A control approach for a three-phase, dual-stage grid-tied photovoltaic (PV) system using predictive control. A forecast current A suggested control mechanism, VS-INC/PCC, is used in the first stage to quickly and correctly track the MPP^10^. A complete sliding mode control approach for a maximum power point tracking MPPT voltage-oriented loop. The goal is to improve performance when used independently^11^. The current paper describes a unique multi-trial vector-based sine cosine algorithm (MTV-SCA) for determining unknown parameters in proton exchange membrane fuel cells (PEMFCs). The tremendous nonlinearity and complexity of the polarization properties of PEMFCs provide major obstacles to precisely predicting these parameters^12^. The goal of this study is to extract the maximum power point. To do this, a sliding mode-based control mechanism for a maximum power point tracking (MPPT) controller was created. The proposed MPPT has been designed and verified on a PEMFC system with a boost controlled by the MPPT and supplying a resistive load^13^. A review of the electrolysis of water by PEMs was released, with a section on modeling PEM water electrolysis^14^. A thorough evaluation of lower-temperature electrolysis design concepts, encompassing alkaline and PEM technology solutions^15^. Furthermore, the classified models were examined according to the mathematical modeling or physical spheres utilized. In^16^, it was discovered that integrating the PV system’s duration and peak power generation with the voltage range of the PEM electrolyzers enhanced hydrogen generation by 12% for a solar-powered PV/PEM electrolyzer able to create sufficient hydrogen for a fuel cell vehicle. In^17^, the results of Modelling and experimentation with a PV-powered PEMEZ were compared. However, experimental research on green hydrogen electrolysis, particularly comparative assessments of AL and PEM systems, is still restricted, impeding progress in both single and hybrid system research and applications. A new way for creating a sliding mode (MPPT) controller for PV systems under rapidly changing atmospheric conditions. Moreover, the typical perturbation and observation, the Genetic method (GAO) is used to find the optimal sliding mode controller (SLMC) gains, which drives the variable step of the Pb&O method^18^. A new drone squadron optimization (DSO) technique that identifies the maximum global power point during PSCS challenges. The study compares particle swarm optimization (PSO), cuckoo search algorithm (CUSA), and grey wolf optimization (GWO) in different operating settings.to affirm the superiority of the proposed technique^19^. A system that uses a proportional-integral-derivative controller, a neural network-equipped grid, a charging station with a Dragon Fly Optimization Algorithm to create electricity, and a maximum power point tracking controller. To optimize power management at the charging station^20^.A novel Hippopotamus Algorithm (HA) for MPPT in solar PV systems with DC microgrids. The performance of HAs is compared to three proven optimization algorithms: Grey Wolf Optimization, Cuckoo Search Algorithm, and Particle-Swarm Optimization, under various operating situations and partial shading conditions. Results show that the HA outperforms conventional approaches in terms of both power output and response time^21^.Clean energy-powered EV charging station using solar energy, standby battery systems, neural network-integrated grids, the enhanced Cuckoo Search Algorithm for Maximum Power Point Tracking, and the Proportional-Integral-Derivative controller^22^. In^23^, a PV power system with high efficiency and compact architecture is described. Solar power conversion microgrids can employ a variety of DC-DC converters. Mismatches between operational load characteristics and PV module arrays provide substantial challenges in PV systems, resulting in Environmental factors, such as temperature fluctuation and sun irradiation, which can significantly reduce efficiency and prevent optimal power output^24^. Fortunately, the MPPT algorithm can alleviate this issue by maintaining the PV array’s maximum power point^25^. Developed a PV system with MPPT and a DC-DC boost converter with self-predictive incremental conductance. The authors’ MATLAB/Simulink simulations show that their technique outperforms the usual incremental conduct (I&C) methodology and produces low ripple output power^26^. In^27^, the FO-PID controller is an expansion of the PID controller with two extra tuning factors: integral value and differential value. Additionally, these parameters increase flexibility in satisfying controller design requirements. A variety of research shows that FO-PID controllers outperform conventional controllers in industrial operations, such as greenhouse and reactor temperature^28^. The suggested fuzzy logic controller updates the control signal instead of employing static PI controller settings. Many optimization strategies have been created to help tune the PI controller settings^29^. This work presents a fractional-order PI controller with meta-heuristic methods to optimize performance and fine-tune parameters and uses a proportional and integral (PI) controller using meta-heuristic approaches to maximize performance and refine parameters. The goal is to optimize the FO-PI controller using meta-heuristic techniques. (PSO, DIFO, GWO). Optimization challenges aim to determine an objective function’s maximum or minimum value. Therefore, it can be an effective tool for controller design. Table 1 highlights the literature on green hydrogen fueled by natural systems and P&O MPPT with different control techniques.

**Table 1:** Highlights the literature on green hydrogen fueled by natural systems and P&O MPPT with different control techniques.

Ref.	Program/methodology	Contribution	Comment/drawbacks
^6,7^	Techno-economic and carbon analysis utilizing genetic algorithms for AWE systems MATLAB/Simulink	Suitable for industrial applications and enhanced electrolyzer capacity for solar/wind hybrid systems.	Large production cost (8.87 $/kg) and emphasizes sizing over dynamic control solutions.
^8^	Comparative techno-economic study of photoelectrochemical (PEC) and photovoltaic-electrolyzer (PV-E) systems based on LCOH (Levelized Cost of Hydrogen) metrics. MATLAB/Simulink	PV-E is more commercially feasible ($6.22/kg) than PEC ($8.43/kg), and its flexibility is highlighted by the ability to size and tune components individually.	Commercial Barriers: It was concluded that PEC lacks successful commercialization prospects owing to catalyst instability. Operational Focus: The research is solely economic, and it does not look at dynamic control or MPPT optimization for the PV-E system.
^9^	Cost modeling and scaling up studies for AEL and PEM electrolysis MATLAB/Simulink	Predicts large CAPEX reductions (up to 30%), identifying 5 MW as a major technical threshold.	Identifies a trade-off between power density and stack longevity and fails to address operational automation and service expenses.
^10^	Dual-stage control: VS-INC/PCC for MPPT, PS-VOC with SVM for integrating into the grid. MATLAB/Simulink	Rapid MPPT tracking, good stability against irradiation variations, and conformance to IEEE-519 standards.	Does not include Partial Shading Conditions (PSC).
^11^	ISMC stands for MPPT Voltage Coupled Loop MATLAB/Simulink	isolated systems have high precision, decreased oscillations, and fewer power losses.	Chattering effect and great mathematical complexity
^12^	MTV-SCA (Multi-Trial Vector Sine Cosine Algorithm) MATLAB/Simulink	High accuracy (98.5% efficiency), prevents voracious convergence, and adjusts to pressure and temperature fluctuations.	Great computational cost for big systems, no HIL testing, and sensitive to practical load fluctuations.
^13^	Improved SMC-based MPPT for PEMFC structure. MATLAB/Simulink	Fast responsiveness, excellent tracking precision, and strong durability under temperature and water content fluctuations.	Initially proven on resistive loads, it requires careful tweaking to reduce the chattering effect.
^14^	Techno-economic study and LCOH examination of PEC and PV-E systems MATLAB/Simulink	Emphasizes PV-E adaptability, cheaper manufacturing costs, and modular optimization possibilities.	PEC demonstrates catalyst instability, high material costs, and a lack of economic practicality.
^15^	A detailed analysis and definition of electrolysis system modeling MATLAB/SIMULINK	Clearly summarizes state-of-the-art and emphasizes spatiotemporal dynamics.	90% of models lack control-oriented descriptions, with a tiny attention to systemic approaches and diagnostic tools.
^16^	Simulation of PV/T and CPV/T-PEM using MPPT MATLAB/Simulink	CPV/T generates more hydrogen, while PEM is suited for varying loads.	Higher sunlight reduces efficiency, and there is no experimental cost analysis.
^17^	Modeling of 1 kW PEM stack using Arduino MEGA controller MATLAB/Simulink	Accurate water transport study with minimal error margin (2.2%) compared to commercial data.	relies on projected characteristics, lacks sophisticated control principles, and neglects hydraulic losses.
^18^	GAO-tuned Sliding Mode with variable step P&O for electric vehicle charging MATLAB/Simulink	Superior stability of the DC bus voltage, rapid calculation, and efficient GMPP tracking.	Specific to small-scale electric vehicle stations and it needs hardware validation and energy storage implementation.
^19^	Drone Squadron Optimization (DSO) for DC microgrids MATLAB/Simulink	Low computational overhead, quick MPP tracking under partial shade, and allows bidirectional flow.	Real-time hardware validation is lacking, and interoperability with multi-phase grids has yet to be solved.
^20^	Dragonfly Optimization PI and Neural Networks for chargers for electric vehicles MATLAB/SIMULINK	Stable DC bus voltage adjusts to five various operating modes, including battery storage.	Incredibly intricate tuning; completely based on simulation, and no experimental or economic analysis offered.
^21^	Solar panels are integrated with a PEM electrolyzer utilizing a DC/DC Buck converter and a typical PID controller. MATLAB/Simulink	Solar panels are integrated with a PEM electrolyzer utilizing a DC/DC Buck converter and a typical PID controller. Experimental Validation: Lab testing validated the results (7.2 ml/min), Low-cost implementation with standard parts, and Matches simulation and experimental data.	The steady-state concept explicitly ignores nonlinear behavior, making it inappropriate for rapid dynamic changes. Basic Control: PID is less effective than advanced controllers (SMC/MPC) in addressing system uncertainties. Low manufacturing scale.
^22^	Cuckoo Search Algorithm (CSA), PID, and Neural Networks for EV charging stability MATLAB/Simulink	Reliable DC bus voltage responds to varied charging demands via NN, and is tested in five scenarios.	Difficult hybrid control logic, lacks real-time hardware inspection, and has high computational expenses for neural networks.
^23^	Comparative Study of Buck, Boost, and Buck-Boost Converters using MPPT MATLAB/Simulink	Buck-Boost was shown to be the most effective for maximal PV power extraction in all situations.	Buck-Boost is more expensive and complex, and it lacks sophisticated, robust control for specific dynamic loads (PEM).
^24^	Efficiency comparison of four converters (Buck, Boost, etc.) utilizing P&O MPPT. MATLAB/Simulink	Non-inverting Buck-Boost had the maximum efficiency (95%).	Used simple P&O with oscillations; evaluated on a very minimal power scale (mW); simulation-only.
^25^	Study of P&O and INC MPPT algorithms with ramping irradiance and changing temperature MATLAB/Simulink	INC delivers more voltage than P&O, and both perform efficiently under constant weather conditions.	Tracking efficiency decreases dramatically under rapid irradiance changes, and P&O displays a delayed reaction, whereas INC shows quick/unstable transitions.
^26^	Improved Simplified InC (SPInC) has a configurable step size (Dd) and no division operations. MATLAB/Simulink	Decreased power ripple, simpler implementation, and quick detection of unexpected weather changes	still relies on predetermined area restrictions, and panel degradation impacts are not explored. Mostly simulation-based.
^27^	Modeling of a 1 kW PEM stack using MATLAB and an Arduino MEGA controller	High accuracy in water transport analysis; low inaccuracy (2.2%) when compared to commercial data.	relies on projected parameters; lacks sophisticated control (MPPT); disregards hydraulic and thermal geometries
^28^	FOFP-FOPID controller improved using hybrid ABC-GA for robotic trajectory tracking MATLAB/Simulink	Highly resistant to external disturbances, flexible tuning, and beats typical PID/FPID.	lacks analytical stability analysis, significant computational cost, and simulation-only (no hardware)
^29^	Takagi-Sugeno (T-S) fuzzy model, featuring an observer-based direct MPPT controller. MATLAB/Simulink	Eliminates the requirement for MPP searching and radiation sensors, enables stringent stability analysis, and is resilient to perturbation.	Great mathematical complexity (LMI-based design), and heavily reliant on the correctness of fuzzy model rules

### Contribution

Employing the PV array as a direct DC power source to produce hydrogen, this study proposes a unique combination of photovoltaic (PV) systems and proton exchange membrane (PEM) electrolyzer. To maximize the generation of energy and hydrogen yield, the study is divided into two parts. The first section examines several control techniques and maximum power point tracking (MPPT) algorithms for optimizing PV system output power in dynamic environmental situations. In the second stage, the PEM electrolyzer is tested under various temperature situations to identify the optimal working parameters for hydrogen generation. This two-phase strategy provides a comprehensive framework for increasing the efficiency of solar-powered hydrogen-generating devices.

### Methodology

1. In this study, a solar system with P&O MPPT and certain control systems, such as PI and FOPI, was investigated, and their parameters were regulated by different algorithms such as DCSO, PSO, and GWO. Additionally, a fuzzy logic controller was investigated.
2. Then, this type was utilized to power a PEM Electrolyzer. Each portion was examined independently, and comparisons were performed according to the instructions below.
3. Comparative performance analysis of a PV system with different settings under a range of temperatures from 5 °C to 100 °C and irradiation conditions from 1000 W/m^2^ to 500 W/m^2^.
4. Illustrate A comparative analysis of the variety of DC/DC converters (Buck, Boost, Buck-Boost) utilized in PV solar systems connected to a DC load (electrolyzer). Using the P&O approach, an MPPT controller regulates the converter’s duty ratio. To verify the viability of the suggested model.
5. The focus of this study is on modeling and simulation of photovoltaic systems supplying a PEM electrolyzer for hydrogen production. The results are compared with those of various PV cases regarding the amount of hydrogen generated and the efficiency of the electrolyzer. Next, examine how altering the electrolyzer’s temperature between 25 °C and 65 °C affects the electrolyzer’s efficiency and the quantity of hydrogen it produces.

## Proposed system

**Fig. 2:**
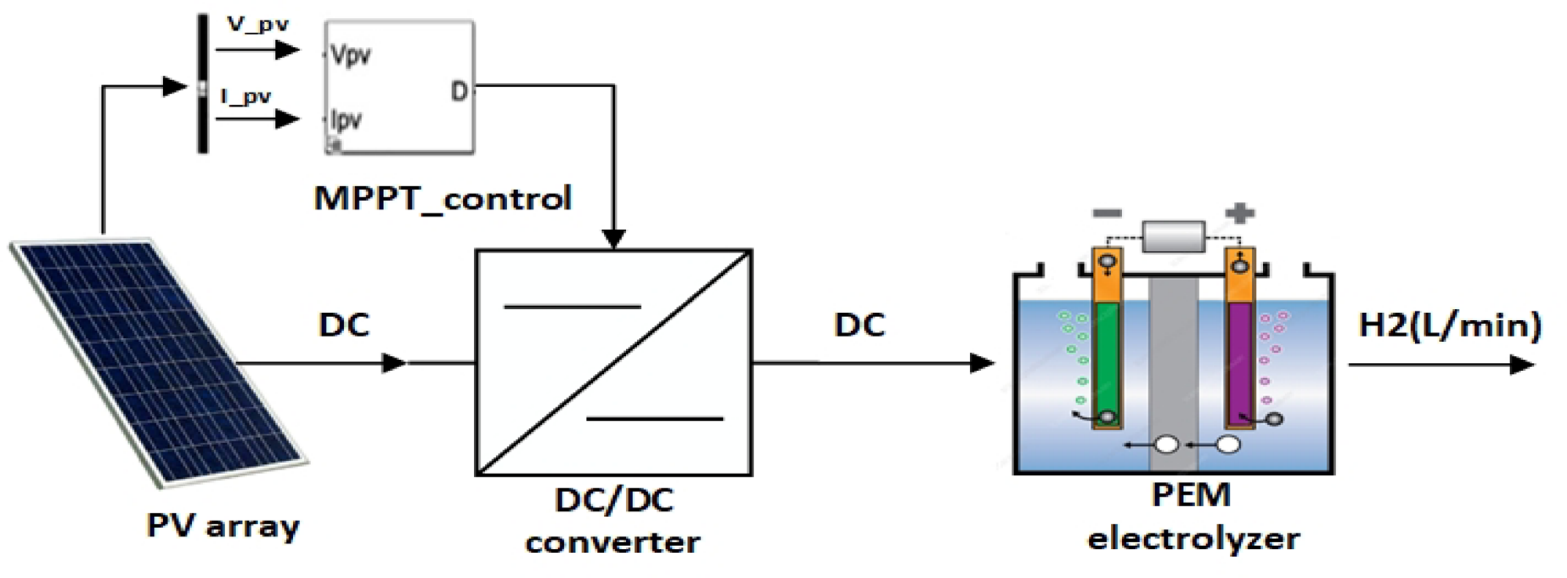
Proposed system for manufacturing hydrogen using a PV system.

The system under examination is made up of four primary components. The first component is a photovoltaic array that generates direct current power. The following section is a DC/DC converter, which regulates the quantity of DC energy that passes to the electrolyzer. The third part is an electrolyzer, which serves to separate water into hydrogen and oxygen, and with its help, this work aims to measure the amount of hydrogen generated. The final part is MPPT techniques, which are used to track and modify the operating conditions of the solar energy system to ensure that it operates at or near the maximum power point, as shown in Fig. 2.

### PV-modeling

**Fig. 3:**
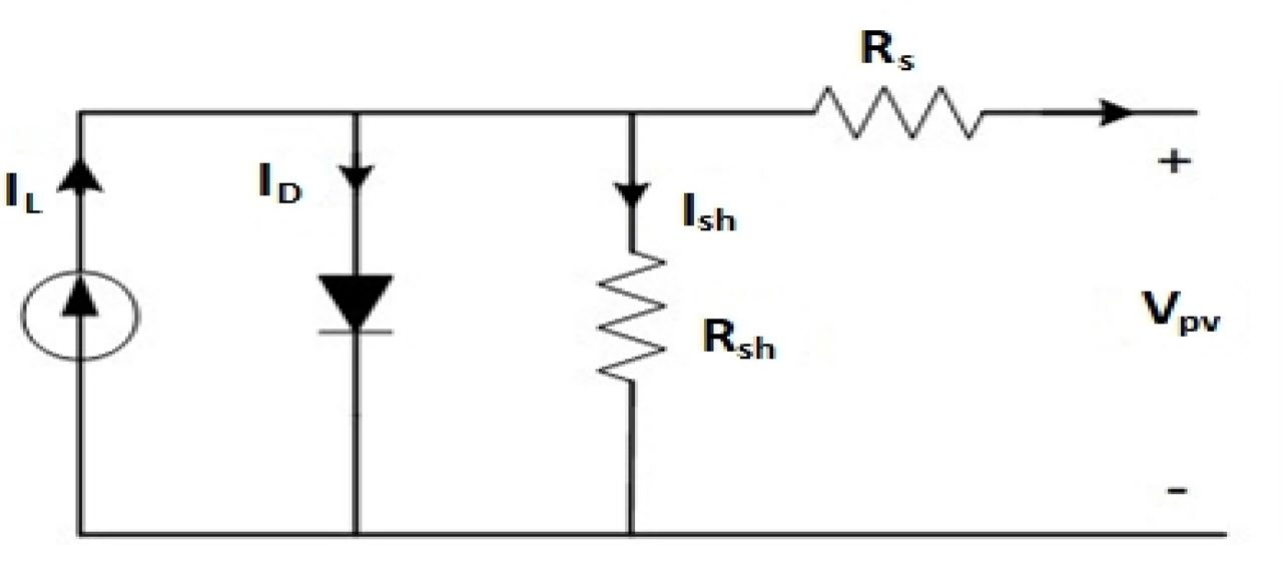
Shows the equivalent circuit of a single PV cell.

A photovoltaic system is made up of a single panel or a set of panels that are coupled together to generate a specific amount of power. The panels listed above are made up of solar cells. This model’s design took temperature and solar radiation into account. The maximum output power of the cell is anticipated via scaling to a reference measurement, followed by an interpolation method on the (V-I) curves that describe the photovoltaic cell’s performance at all operational points^33^. Offer the most common model of the solar cell addressed in this research, using simple calculations based on the manufacturer’s specification data. Figure 3 depicts an analogous circuit that models cell performance dependent on solar radiation and temperature, with four components (current source, diode, parallel resistor $\:{\boldsymbol{R}}_{\boldsymbol{s}\boldsymbol{h}}$, and a series resistor $\:{\boldsymbol{R}}_{\boldsymbol{s}}$)^34^. Since the voltage and current produced by the photovoltaic cell are proportional to solar radiation, and adopting the diode model, they decline to zero in darkness. The diode expresses this behavior, and the net current created by the cell may be estimated using Eq. (1) as shown below.

1 $$\:{\boldsymbol{I}}_{\boldsymbol{p}\boldsymbol{v}}={\boldsymbol{I}}_{\boldsymbol{l}}-{\boldsymbol{I}}_{\boldsymbol{D}}-{\boldsymbol{I}}_{\boldsymbol{s}\boldsymbol{h}}$$

Where ($\:{\boldsymbol{I}}_{\boldsymbol{l}}$) is the cell light-generated current caused by the photo effect, $\:\left({\boldsymbol{I}}_{\boldsymbol{s}\boldsymbol{h}}\right)$ is the shunt leakage current, and $\:\left({\boldsymbol{I}}_{\boldsymbol{D}}\right)$ Is the diode current defined by Eq. (2)

2 $$\:{\boldsymbol{I}}_{\boldsymbol{D}}={\:\boldsymbol{I}}_{0}({\boldsymbol{e}}^{\left(\frac{\boldsymbol{q}\left({\boldsymbol{V}}_{\boldsymbol{p}\boldsymbol{v}}+{\boldsymbol{I}}_{\boldsymbol{p}\boldsymbol{v}}\boldsymbol{*}{\boldsymbol{R}}_{\boldsymbol{s}}\right)}{\:{\boldsymbol{A}}_{\boldsymbol{p}\boldsymbol{v}}\boldsymbol{*}{\boldsymbol{K}\boldsymbol{*}\boldsymbol{T}}_{\boldsymbol{p}\boldsymbol{v}}}\right)}-1\:\:\:\:$$

In this equation $\:\left({\boldsymbol{V}}_{\boldsymbol{p}\boldsymbol{v}}\right)$ is the PV cell output Voltage, ($\:{\:\boldsymbol{I}}_{0}$) is the diode saturation current, (q) is the electron charge, (K) is the Boltzmann constant (1.3806*10–23 J/K), ($\:{\:\boldsymbol{T}}_{\boldsymbol{p}\boldsymbol{v}})$ Is the PV cell temperature in Kelvin, and ($\:{\:\boldsymbol{A}}_{\boldsymbol{p}\boldsymbol{v}})\:$Is the ideality coefficient of the PV cells and the shunt leakage.

The current through the shunt resistor is determined by Eq. (3)

3 $$\:{\boldsymbol{I}}_{\boldsymbol{s}\boldsymbol{h}}=\left(\frac{{\boldsymbol{V}}_{\boldsymbol{p}\boldsymbol{v}}+{\boldsymbol{I}}_{\boldsymbol{p}\boldsymbol{v}}\boldsymbol{*}{\boldsymbol{R}}_{\boldsymbol{s}}}{{\boldsymbol{R}}_{\boldsymbol{s}\boldsymbol{h}}}\right)$$

The PV system represented in this study is constructed of 12 PV modules of Sun Earth Solar Trina solar TSM-350DEG 14.14(II) type and was linked as follows: 2 modules per string, 10 strings connected in parallel. Table 2 displays statistics from the used PV arrays, which are derived from^35^. As explained in the next section, the PEMEZ uses a DC/DC converter attached to its output.

### PEM electrolyzer modelling

**Fig. 4:**
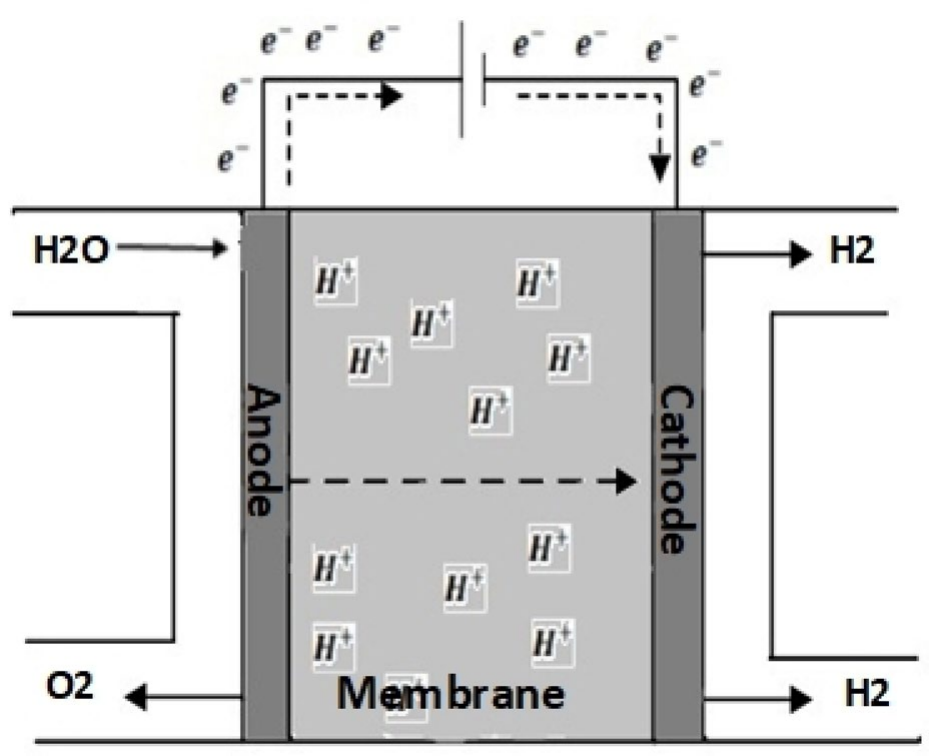
Schematic of the PEM electrolyzer.

Water is the most abundant source of hydrogen, which may be created using a method known as water electrolysis. This involves running DC current through two electrodes submerged in water to break the water molecule into hydrogen and oxygen. William Nicholson and Sir Anthony Carlisle devised this method around 1800. Electrolysis is the most promising method for generating hydrogen from renewable sources. It can generate hydrogen with no emissions. Using only water in the process results in 99.9995% pure hydrogen and oxygen. An electrolyzer (EZ) is a device that performs electrochemical processes using a stack of cells. The most widely utilized commercial electrolyzer technology is alkaline and polymer electrolyte membrane (PEM) electrolyzers. It may also have been referred to as Proton Exchange Membrane Electrolyzers (PEMEZ) according to the description of chemical reactions that occur across the membrane^30^. PEMEZ was chosen for this investigation because of its special characteristics, such as the use of a solid polymer membrane (hence the name). A perfluorinated sulfonic acid polymer, commonly referred to as Nafion, serves as the electrolyte for this membrane. The PEMEZ has pressures between the atmosphere and 30 bar. It operates at a temperature below 85 °C and has an efficiency of 65 to 80%^31^. PEMEZ uses water as an electrolyte and two polarized electrodes composed of platinum conductors, which are chemically inactive (see Fig. 4). The chemically inactive electrodes prevent undesired interactions with hydrogen or oxygen ions^32^. When current flows over a membrane, positive charge carriers, such as hydrogen ions, pull negatively charged cathodes, while positively charged oxygen ions pull anodes.

Equations (4) and (5) illustrate the processes at the anode and cathode of a PEMEZ.

The reaction occurring at the anode:

4 $$\:\:{\boldsymbol{H}}_{2}\mathbf{O}\:\:\to\:\:{2\boldsymbol{H}}^{+}+\:\frac{1}{2}{\boldsymbol{O}}_{2}+2{\boldsymbol{e}}^{-}$$

The reaction occurring at the cathode:

5 $$\:{\:\:\:2\boldsymbol{H}}^{+}+2{\boldsymbol{e}}^{-}\to\:\:\:{\boldsymbol{H}}_{2}\:\:$$

They may be combined into one equation representing the whole reaction, as shown in Eq. (6).

6 $$\:{\boldsymbol{H}}_{2}\mathbf{O}\:+\mathbf{E}\mathbf{n}\mathbf{e}\mathbf{r}\mathbf{g}\mathbf{y}\to\:\:{\boldsymbol{H}}_{2}\:+\:\frac{1}{2}{\boldsymbol{O}}_{2}$$

The energy in Eq. (6) refers to the electric energy given to the EZ for the electrolysis process, which will be detailed in the following section.

The enthalpy from creation (∆H=285.84 kJ/mol) in Eq. (6) can be divided into thermal energy (∆S kJ/mol.K) multiplied by operational absolute temperature (T) and helpful work (∆G kJ/mol), which can be represented as electrical energy. However, ∆G is limited by the second term. So, the total energy is computed using Eq. (7).

7 $$\:\varDelta\:\boldsymbol{H}=\varDelta\:\boldsymbol{G}+\boldsymbol{T}.\varDelta\:\boldsymbol{S}$$

The change in enthalpy can be caused by a change in either the first term (∆G) or the second term (T*S), or both simultaneously. However, the change in the PEMEZ is only caused by an electrical change (∆G). So ∆H = ∆G. The voltage required for the electrolyzer cell may be estimated using Eqs. (8) and (9). These are referred to as reversible, standard, or ideal voltage, and the Nernst equation, accordingly^33^.

8 $$\:{\boldsymbol{V}}_{\boldsymbol{i}}=\:\frac{\varDelta\:\boldsymbol{G}}{2\boldsymbol{F}}\:$$

9 $$\:{\boldsymbol{e}}_{\boldsymbol{r}\boldsymbol{e}\boldsymbol{v}}=\:{\boldsymbol{V}}_{\boldsymbol{i}}+\:\frac{\boldsymbol{K}\boldsymbol{T}}{2\boldsymbol{F}}\mathbf{ln}\left(\frac{\:{\boldsymbol{P}\boldsymbol{H}}_{2}\:{{\boldsymbol{P}\boldsymbol{o}}_{2}}^{0.5}}{\boldsymbol{P}{\boldsymbol{H}}_{2}\boldsymbol{o}}\right)$$

Where (F) is the Faraday constant, and its value is 96,487 C/mol, V_i_ is the ideal voltage, its value is 1.233 V under nominal working conditions (20 °C, 1 atm pressure). To overcome losses such as activation, ohmic, and concentration, the voltage applied to the electrolyzer must be higher than the ideal voltage to initiate the electrochemical reaction and achieve the desired hydrogen production. Commercial PEMEZ devices exhibit a linear polarization curve (voltage and current) with a constant slope. The EZ characteristic can be expressed as a linear relationship between input voltage and current. This relationship is analogous to electrical resistance performance. This principle may be explored and applied to the model. The electrical response is approximated using a DC voltage source and a series of linked electrical resistances. The PEMEZ zero-current voltage is provided by a DC voltage source and varies with input current due to series resistance. The voltage source and resistance values must be calculated using the PEMEZ polarization curve. These numbers describe the model parameters that need to be updated to align with PEMEZ’s features. Additionally, this model considers the impact of temperature and stack pressure variations. The model has been constructed to provide a polarization curve that resembles commercial PEMEZ device performance under various operating situations without requiring any changes to the model parameters^34^. The voltage response of this model (VEZ) is computed based on two^34,35^. As indicated in Eq. (10). The first term expresses the zero-current voltage, whereas the second term is dependent on input current, and both terms are affected by operational temperature and pressure.

10 $$\:{V}_{EZ}\left(T,P\right)={e}_{rev}\left(T,P\right)+\:{I}_{EZ}{R}_{i}(T,P)$$

**Fig. 5:**
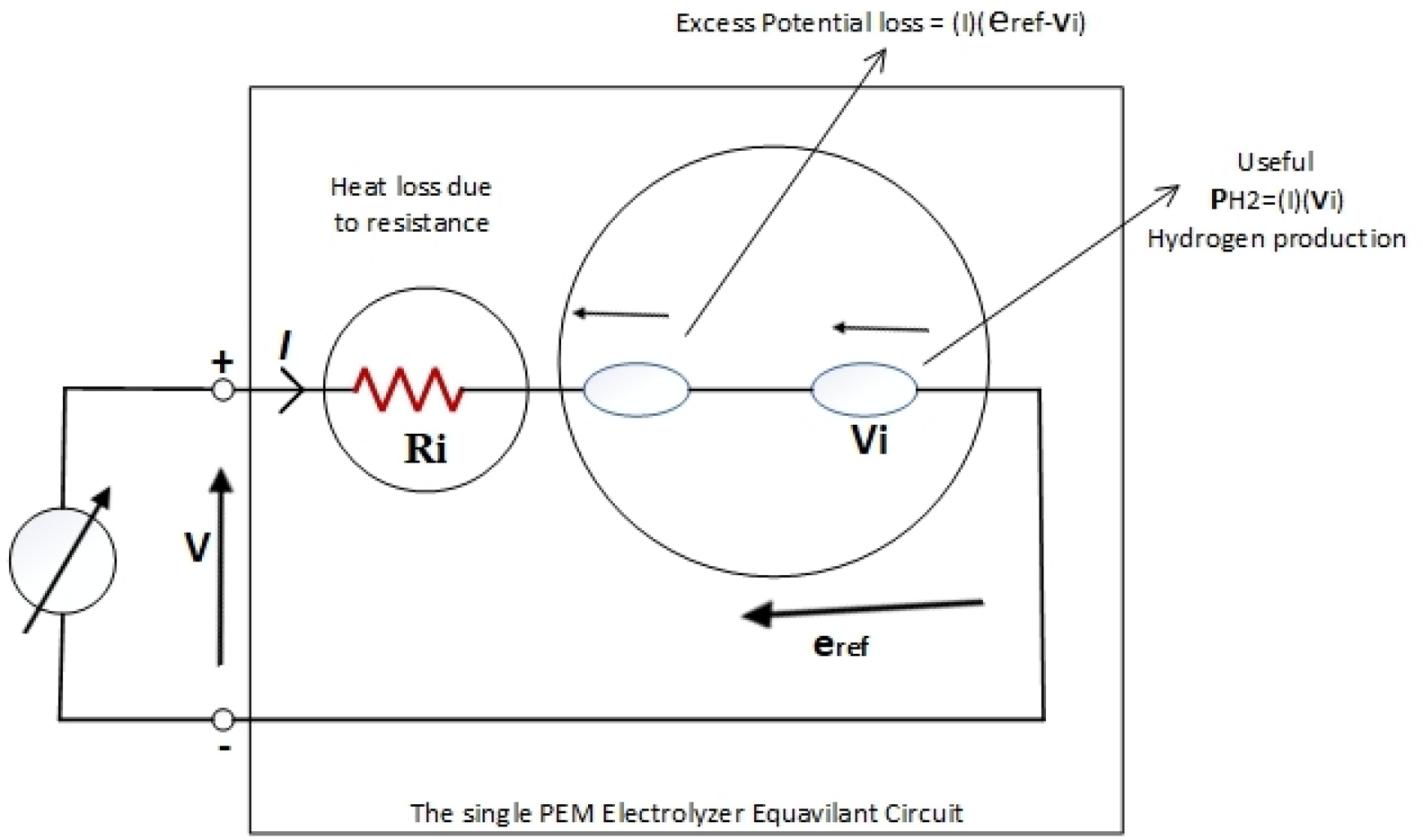
The equivalent circuit model for a single cell PEM electrolyzer.

This simple PEMEZ model has been created using the basis of the reversible potential $\:{e}_{rev}$, incorporating the ideal voltage, V_i_, the internal resistance of the device R_i_, in addition to terms indicating the voltage loss within the polymer electrolyte membrane. As shown in Fig. 4, which illustrates the physics of the suggested model of the PEMEZ expressed in current ($\:{I}_{EZ}$) changes according to the altered input voltage $\:{V}_{EZ}$^36^ (Fig. 5).

The PEMEZ membrane’s reversible potential varies moderately with operational temperature and pressure (P and T). Equations (11) and (12) can be used to calculate reverse voltage and internal resistance.

11 $$\:{\boldsymbol{e}}_{\boldsymbol{r}\boldsymbol{e}\boldsymbol{v}}\left(\boldsymbol{T},\boldsymbol{P}\right)={\boldsymbol{e}}_{\boldsymbol{r}\boldsymbol{e}\boldsymbol{v}^\circ\:}+\frac{\boldsymbol{R}\boldsymbol{T}}{2\boldsymbol{F}}\mathbf{ln}\left(\frac{\boldsymbol{P}}{\boldsymbol{p}^\circ\:}\right)\:\:$$

12 $$\:{\boldsymbol{R}}_{\boldsymbol{i}}\left(\boldsymbol{T},\boldsymbol{P}\right)={\boldsymbol{R}^\circ\:}_{\boldsymbol{i}}+\:{\boldsymbol{K}}_{\boldsymbol{E}\boldsymbol{Z}}\mathbf{ln}\left(\frac{\boldsymbol{P}}{\boldsymbol{p}^\circ\:}\right)\:+\:{\boldsymbol{d}\boldsymbol{R}}_{\boldsymbol{i}}(\boldsymbol{T}-\boldsymbol{T}\boldsymbol{o})$$

Where$\:\:{\boldsymbol{e}}_{\boldsymbol{r}\boldsymbol{e}\boldsymbol{v}^\circ\:}$ is The reverse voltage at reference temperature (T°), membrane pressure (p°), and the optimal gas constant (R) are all stated in J/mol.°K, R_i_°, $\:{K}_{EZ}\:$and dRt denote the internal resistance (ohm), curve fitting parameters (V/A), and resistance coefficient of temperature (ohm/°K), respectively. The settings were reviewed and altered to imitate the commercial device’s properties, as described in^37^. To get the total voltage applied across the stack dynamic model, multiply the PEMEZ voltage in Eq. (10) by the number of series cells ($\:{N}_{cellsEZ}$) in the PEMEZ stack. The quantity of hydrogen gas generated by the PEMEZ in ( mol/s) may be calculated from Eq. (13) using the cell current, which relies on the pressure and the temperature of the cell. This amount is also proportional to the number of series cells $\:{N}_{cellsEZ}$ of the PEMEZ stack.

13 $$\:{\boldsymbol{V}}_{\boldsymbol{H}2}={\boldsymbol{N}}_{\boldsymbol{c}\boldsymbol{e}\boldsymbol{l}\boldsymbol{l}\boldsymbol{s}\boldsymbol{E}\boldsymbol{Z}}\left(\frac{{\boldsymbol{I}}_{\boldsymbol{E}\boldsymbol{Z}}}{2\boldsymbol{F}}\right)$$

The electrochemical energy per second $\:{P}_{H2}$ generated by chemical reactions within the PEMEZ, which corresponds to the quantity of hydrogen generation $\:{V}_{H2}$ It is represented by the usable power generated in the form of hydrogen gas. Equation (14). Where the ideal voltage( $\:{V}_{i}$) can be determined as described in Eq. (11).

$$\:{\boldsymbol{V}}_{\boldsymbol{i}}=\:\frac{\varDelta\:\boldsymbol{H}}{2\boldsymbol{F}}$$

14 $$\:{\boldsymbol{P}}_{\boldsymbol{H}2}={\boldsymbol{I}}_{\boldsymbol{E}\boldsymbol{Z}}{\boldsymbol{V}}_{\boldsymbol{i}\:\:\:}$$

## Optimization problem

### Fitness function

Turning the PI controller parameters ($\:{\boldsymbol{K}}_{\boldsymbol{p}}$,$\:{\boldsymbol{K}}_{\boldsymbol{i}}$ ) to accomplish the objective function in Eq. (15).

15 $$\:\boldsymbol{f}\boldsymbol{i}\boldsymbol{t}\boldsymbol{n}\boldsymbol{e}\boldsymbol{s}\boldsymbol{s}=\mathbf{min}\left(\boldsymbol{I}\boldsymbol{T}\boldsymbol{A}\boldsymbol{E}\right)\:$$

Where fitness is a goal function, and ITSE is the integral time square error, Eq. (16) provides a mathematical expression for the ITSE performance index.

16 $$\:\boldsymbol{I}\boldsymbol{T}\boldsymbol{S}\boldsymbol{E}=\:{\int\:}_{0}^{\boldsymbol{\infty\:}}\boldsymbol{t}{\boldsymbol{e}}^{2}\left(\boldsymbol{t}\right)\boldsymbol{d}\boldsymbol{t}$$

where e is the total error between the actual output voltage of the PV cell and the reference voltage from the P&O MPPT. In this work, we use the differential creative search algorithm as the main optimizer to accomplish the objective function and compare its behavior with other optimizers like PSO and GWO to provide a fair comparison, which will be clearly stated in the results section.

### PI controller

**Fig. 6:**
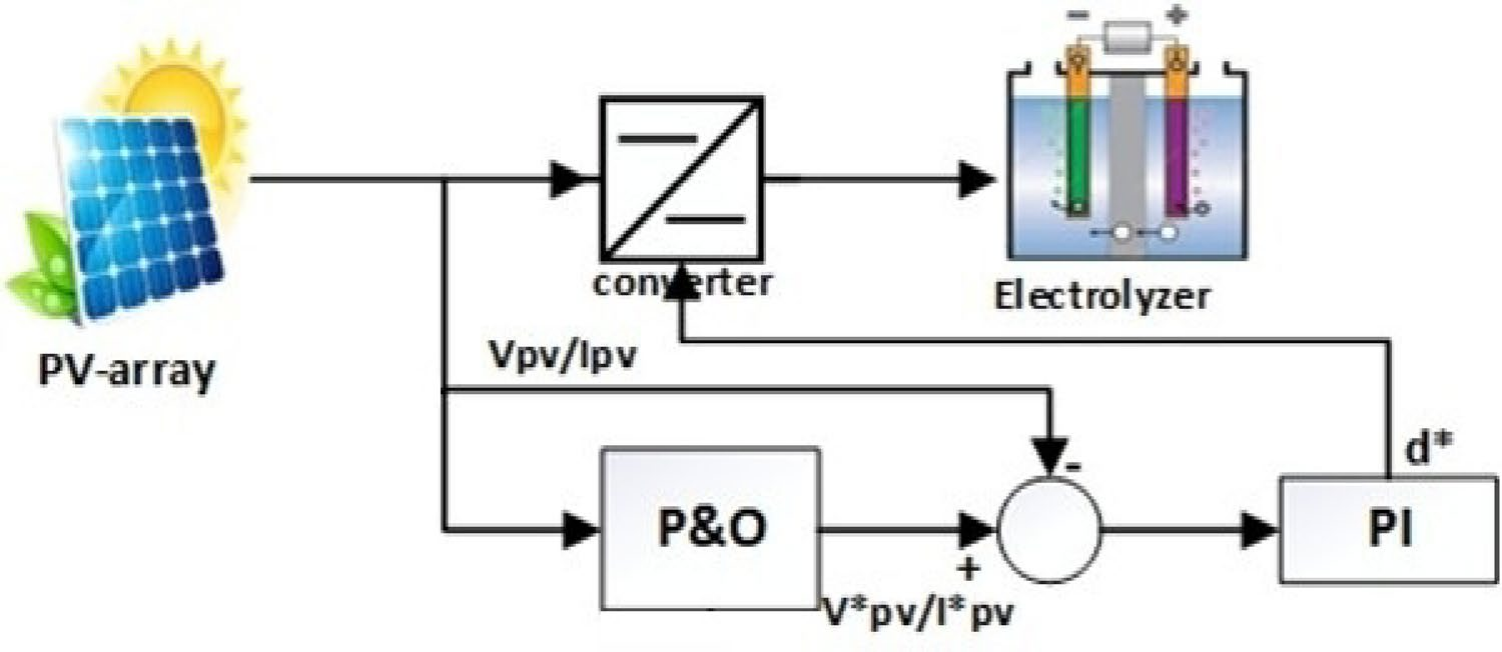
Block diagram of adaptive P&O technique.

The proportional and integral controller generates an outcome signal, u (t), that is proportionate to each the input signals, V_i_ (t), and its integral, V_i_ (t), as shown in Eq. (17) and Fig. 6.

17 $$\:\boldsymbol{u}\left(\boldsymbol{t}\right)={\boldsymbol{K}}_{\boldsymbol{p}}{\boldsymbol{V}}_{\boldsymbol{i}}\left(\boldsymbol{t}\right)+{\boldsymbol{K}}_{\boldsymbol{i}}\int\:{\boldsymbol{V}}_{\boldsymbol{i}}\left(\boldsymbol{t}\right)\:$$

The MPPT yields a reference voltage ($\:{V}_{ref}$). When comparing Vref to the PV voltage (Vpv), an error signal is generated and sent to the PI control. Proper selection of proportional gain (K_p_) and integral gain (K_i_) gives the desired response. When the converter receives electricity from the PV panel and the PI controller begins, it adjusts the duty cycle, which affects the entered value perceived by the controller. Controller tuning involves adjusting parameters to fulfill performance specifications.

The proposed adaptive approach calculates the error between two subsequent array power signals based on observed voltage and current data. Adaptive perturb. Initially, the inaccuracy is significant during hill climbing but reduces as the maximum power operating point approaches steady state. This mistake can be considered as an error signal in a closed-loop system, requiring minimization at steady state. To attain these aims, handle the error signal using a typical PI controller, which is the basis of the suggested approach. This PI controller serves as an adaptive perturb value generator for the reference array voltage.

### Differentiated creative search optimization algorithm (DCSO)

Differentiated teaching, which promotes individualized learning and fosters deep understanding and skill development while promoting student variety, is the source of this differentiated knowledge acquisition^38^. The DCS optimizer maintains a steady population size and treats each individual as a team member. Responsibilities are assigned based on individual performance, aligning with the concepts of differentiated knowledge development. Top performers use divergent thinking to explore, whereas the remainder of the team uses convergent thinking for exploitation, aligning with the creative realism approach. The methodology provides each team member with an individual skill acquisition rate.

**Fig. 7:**
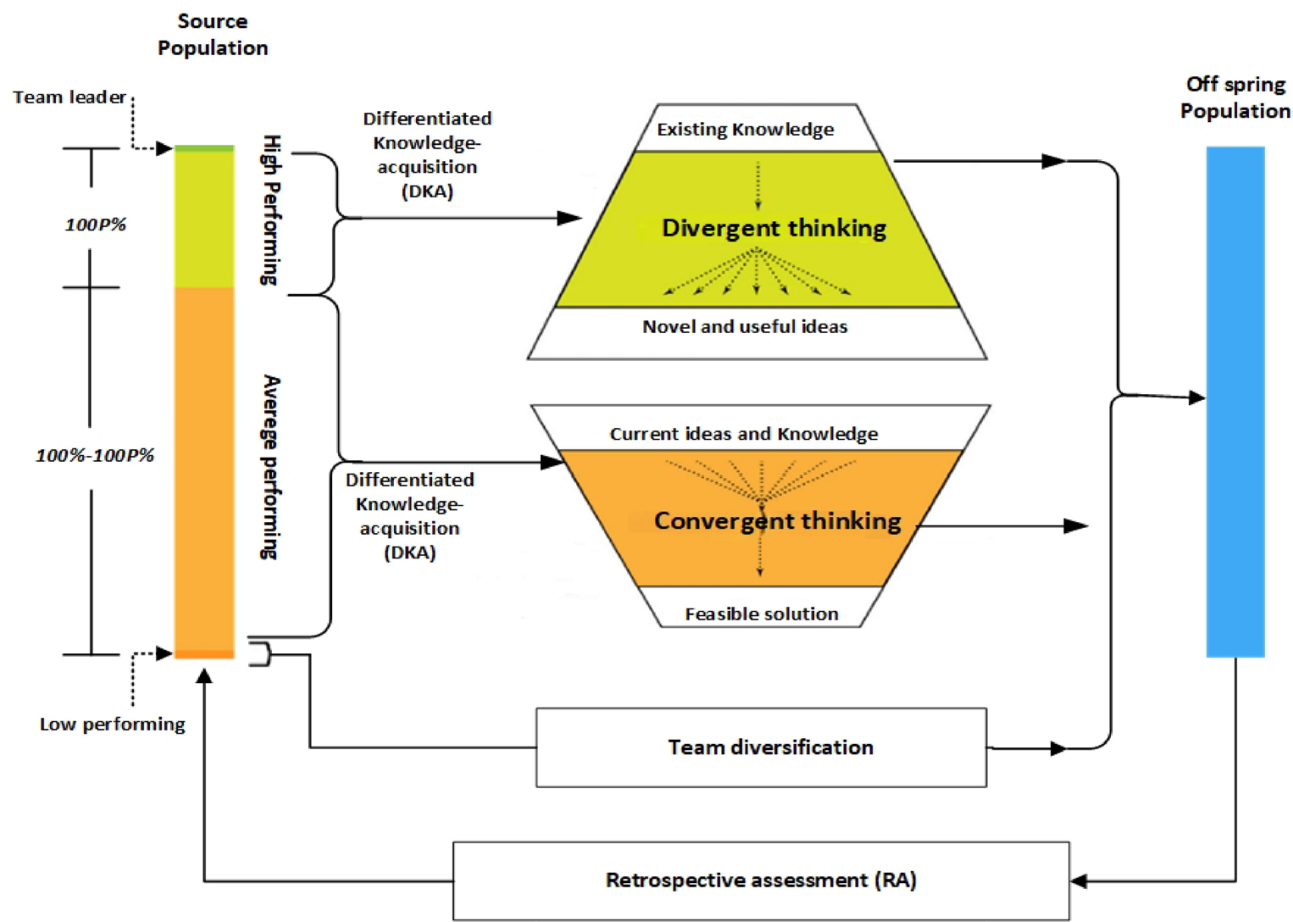
The DCS optimization model.

Based on a given rating, which aligns with the differentiated rate. This systematic approach to knowledge acquisition aligns with the DE cycle’s integration stage and serves a comparable function in our paradigm. The retrospective evaluation assesses each iteration’s outcomes as shown in Fig. 7. DCS assigns distinct roles to team members based on their performance level: top performers create new ideas, moderate High achievers develop ideas into solutions, whereas low performers focus on improving variety among teams. The differential knowledge-acquisition technique improves performance by assessing Unique skill levels and modifying the acquisition rate accordingly. The RA process picks enhanced Individuals over eras and monitors the best performers. The RA process also generates data to track performance. A strategy based on data guides planned actions and increases worker efficiency^38^.

### DCS algorithm implementation

The DCS method begins by randomly initiating one individual to compute fitness and acquire the best value for each parameter across all team members. The optimization phases for the DCS method are detailed below.

#### Step 1

involves defining the algorithm’s parameters, such as the number of populations (N_P_), the upper and lower boundaries (L_B_, U_B_), the maximum number of iterations (NFEmax), and the number of variables (D).

#### Step 1.1

Set the lower and upper bounds restrictions (L_1_, L_2_, L_3_, L_4_, L_5_, L_6_, L_7_, L_8_) and upper bounds restrictions (U_1_, U_2_, U_3_, U_4_, U_5_, U_6_, U_7_, U_8_).

#### Step 1.2

Define PI control variables ($\:{K}_{p}^{v}$, $\:{K}_{i}^{v},\:{K}_{p}^{q}$, $\:{K}_{i}^{q}$, $\:{K}_{p}^{d},\:{K}_{i}^{d},$ $\:{K}_{p}^{Q}and\:{K}_{i}^{Q})$. and create individuals as zeros in the vector based on the total size of the population (N_P_) and the variety of variables.

(D) as below:

18 $$Individual = zero\left[ {\begin{array}{*{20}c} {X_{{1,1}} } & \ldots & {X_{{1,d}} } & {X_{{1,D - 1}} } & {X_{{1,D}} } \\ {X_{{i,1}} } & \vdots & {X_{{i,d}} } & {X_{{i,D - 1}} } & {X_{{i,D}} } \\ \ldots & \ldots & \ldots & \ldots & \ldots \\ {X_{{NP - 1,1}} } & \ldots & {X_{{NP - 1,d}} } & {X_{{NP - 1,D - 1}} } & {X_{{NP - 1,D}} } \\ {X_{{NP,1}} } & \ldots & {X_{{NP,d}} } & {X_{{NP,D - 1}} } & {X_{{NP,D}} } \\ \end{array} } \right]$$

#### Step 2

Set up the individual size. In this stage, the individual population is created with an array containing D*NP, then computed as follows.

19 $$\:{\boldsymbol{X}}_{\boldsymbol{i},\boldsymbol{j}}={\boldsymbol{L}\boldsymbol{B}}_{\boldsymbol{j}}+\boldsymbol{U}\left(\mathrm{0,1}\right)\boldsymbol{*}({\boldsymbol{U}\boldsymbol{B}}_{\boldsymbol{j}}-{\boldsymbol{L}\boldsymbol{B}}_{\boldsymbol{j}})$$

*i* = 1, 2, …….,NP, *j* = 1,2,…….D.

The population vectors’ initialization value is the following:

20 $$individual = \left[ {\begin{array}{*{20}c} {X_{{1,1}} } & \ldots & {X_{{1,d}} } & {X_{{1,D - 1}} } & {X_{{1,D}} } \\ {X_{{i,1}} } & \vdots & {X_{{i,d}} } & {X_{{i,D - 1}} } & {X_{{i,D}} } \\ \ldots & \ldots & \ldots & \ldots & \ldots \\ {X_{{NP - 1,1}} } & \ldots & {X_{{NP - 1,d}} } & {X_{{NP - 1,D - 1}} } & {X_{{NP - 1,D}} } \\ {X_{{NP,1}} } & \ldots & {X_{{NP,d}} } & {X_{{NP,D - 1}} } & {X_{{NP,D}} } \\ \end{array} } \right]$$

#### Step 3

the portion in the j-th location (dimension) of the trial individual $\:{V}_{i,t}$ is updated as follows.

21 $$\user2{V}_{{\user2{i},\user2{j}}} = \user2{w*X}_{{\user2{best},\user2{j}}} + \user2{~\lambda }_{\user2{t}} \user2{*}\left( {\user2{X}_{{\user2{r}2,\user2{j}}} - \user2{~X}_{{\user2{i},\user2{j}}} } \right) + \user2{~\omega }_{{\user2{i},\user2{t}}} \user2{*}\left( {\user2{X}_{{\user2{r}1,\user2{j}}} - \user2{~X}_{{\user2{i},\user2{j}}} } \right)$$

This instance is approached successfully by choosing X_r1_ and X_r2,_ which fit.

ω and λt parameters.

#### Step 4

The Formula for creating a new member is the following.

22 $$\:{\boldsymbol{V}}_{\boldsymbol{N}\boldsymbol{P}}=\boldsymbol{L}\boldsymbol{B}+\boldsymbol{r}\boldsymbol{a}\boldsymbol{n}\boldsymbol{d}\left(.\right)\boldsymbol{*}(\boldsymbol{U}\boldsymbol{B}-\boldsymbol{L}\boldsymbol{B})$$

#### Step 5

Evaluate fitness: The new vector is enhanced based on the aforementioned rule and assessed using the fitness function. Updated as follows:

$$\:\boldsymbol{i}\boldsymbol{f}\:\boldsymbol{f}\boldsymbol{i}\boldsymbol{t}\boldsymbol{n}\boldsymbol{e}\boldsymbol{s}\boldsymbol{s}\left({\boldsymbol{V}}_{\boldsymbol{i},\boldsymbol{j}}^{\boldsymbol{t}+1}\right)<\boldsymbol{f}\boldsymbol{i}\boldsymbol{t}\boldsymbol{n}\boldsymbol{e}\boldsymbol{s}\boldsymbol{s}\left({\boldsymbol{X}}_{\boldsymbol{i},\boldsymbol{j}}^{\boldsymbol{t}}\right)\:\boldsymbol{t}\boldsymbol{h}\boldsymbol{e}\boldsymbol{n}$$

23 $$\:{\boldsymbol{X}}_{\boldsymbol{i},\boldsymbol{j}}^{\boldsymbol{t}}={\boldsymbol{V}}_{\boldsymbol{i},\boldsymbol{j}}^{\boldsymbol{t}+1}$$

end

$$\:\boldsymbol{i}\boldsymbol{f}\:\boldsymbol{f}\boldsymbol{i}\boldsymbol{t}\boldsymbol{n}\boldsymbol{e}\boldsymbol{s}\boldsymbol{s}\left({\boldsymbol{X}}_{\boldsymbol{i},\boldsymbol{j}}^{\boldsymbol{t}}\right)<\boldsymbol{f}\boldsymbol{i}\boldsymbol{t}\boldsymbol{n}\boldsymbol{e}\boldsymbol{s}\boldsymbol{s}\left(\boldsymbol{X}\boldsymbol{b}\boldsymbol{e}\boldsymbol{s}\boldsymbol{t}\right)\:\boldsymbol{t}\boldsymbol{h}\boldsymbol{e}\boldsymbol{n}$$

24 $$\:X\boldsymbol{b}\boldsymbol{e}\boldsymbol{s}\boldsymbol{t}={\boldsymbol{X}}_{\boldsymbol{i},\boldsymbol{j}}^{\boldsymbol{t}}\:\:$$

end.

#### Step 6

The stopping condition is when the number of iterations approaches approaching the highest level allowable; the optimization stops. Alternatively, go to Steps 3, 4, and 5. The suggested optimization approach is shown in the pseudocode, which demonstrates how. Figure 7 depicts the DCS method, which finds the optimal space solution for the best solution. The DCS algorithm is used to search for the optimum values of the PI controller parameters to reduce the error between V_actual_, the PV output voltage, and V_ref_ from the P&O MPPT technique. In this study, we compare this case with other algorithms like PSO and GWO used for tuning PI controller parameters, as shown in the results section.

### Pseudo code for the DCS-based PI controller algorithm

**Algorithm:**
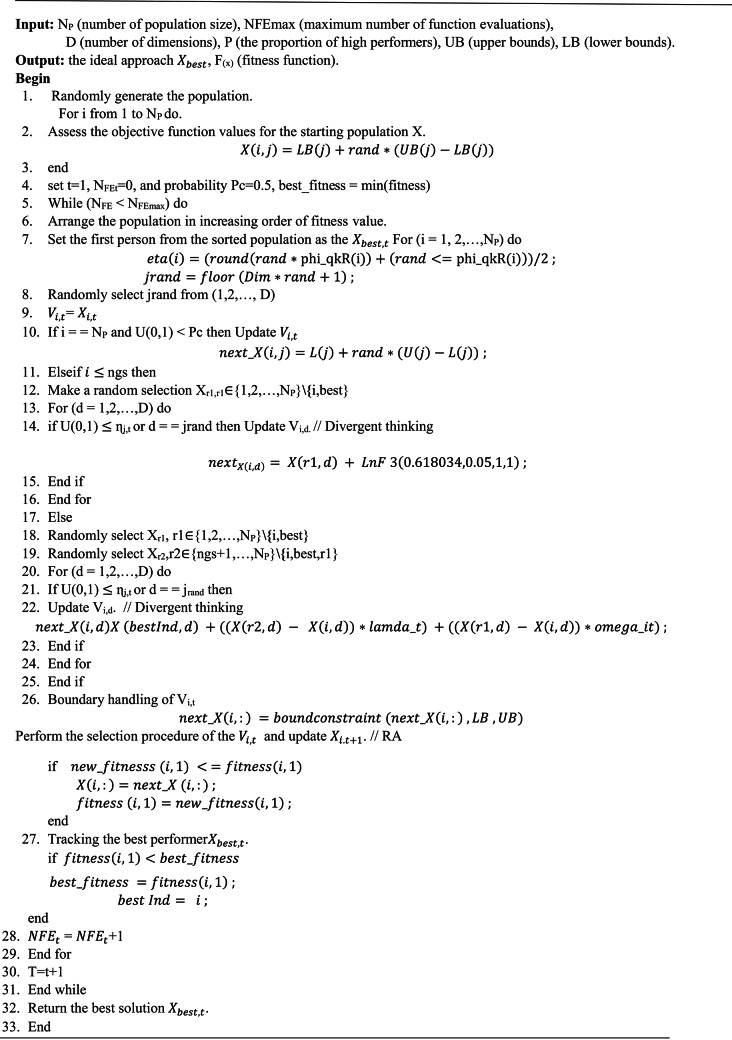
Differentiated creative search (DCS)–based PI controller.

## Results and discussion

### Input data of PV system

A collection of input data was collected and structured to precisely simulate and assess the PV system’s performance. These data contain all the key parameters that characterize system components, ambient conditions, and site-specific features. The primary input values for the analysis are presented in the (Table 2).

**Table 2:** PV array specification data^4^.

Item	Description
Type	Trina Solar TSM-350DEG14.04(Ⅱ)
P_m_	350 W
V_m_	38.5 V
I_m_	9.09
A	(1.984 × 0.998) = 1.98m^2^
No. PV series modules	2
No. of strings	10
Total power of the PV system	7000 W
Input irradiance	1000 W/m^2^

A PV system constructed in MATLAB/SIMULINK version: R2023a, running on AMD Ryzen 7 7435HS, 64-bit operating system, 8 GB RAM laptop. with varying irradiance and temperature levels, and control MPP via P&O MPPT technique with different Controllers. Used to power the PEM electrolyzer, which produces hydrogen. This section is structured into three main parts as follows:

### Scenario-based assessment of PV and PEM systems

In the findings section, I evaluated the solar energy system and proton exchange membrane (PEM) electrolyzer under various operating circumstances. This dual method enabled me to assess each system’s capabilities, efficacy, and limitations in the face of technological and environmental changes. The primary situations I focused on are outlined below:

1. PV production under varying conditions.
2. PEM electrolyzer result under variable conditions.
3. PI controller behavior with three optimizers (DCSO-PSO-GWO).
4. FOPI controller behavior with three optimizers (DCSO-PSO-GWO).
5. Comparison of the Fuzzy logic controller with the previous two controllers.
6. Every scenario provided useful information regarding the behavior of these technologies, both individually and in combination, especially under real-world conditions. In the following parts, I will go over each situation in great depth, focusing on the approach, findings, and observations.

### PV production under varying conditions

#### Constant irradiation and temperature

**Fig. 8:**
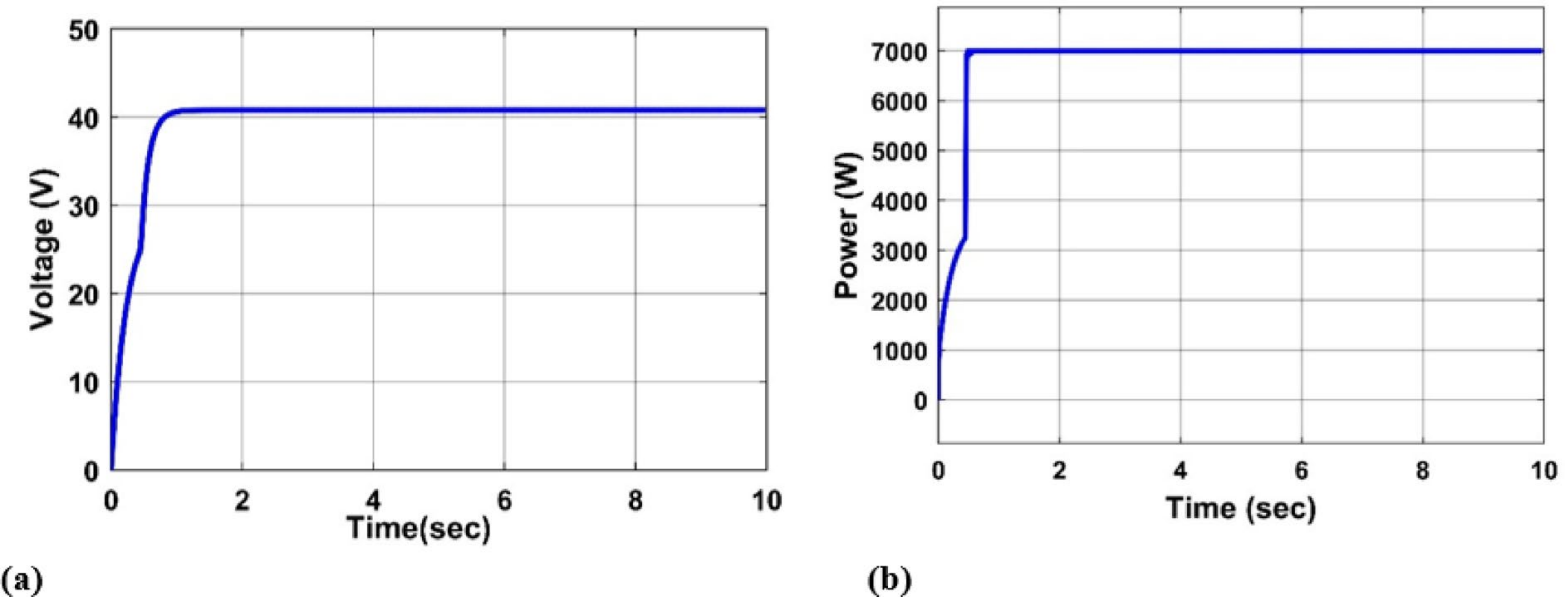
(**a**) PV output Voltage under 1000 W/m2 and 25 C, (**b**) PV output Power under 1000 W/m2 and 25 C.

In this section, we examine the PV system’s voltage and power outputs under 1000 W/m2 radiation and a temperature of 25 °C, as illustrated in Fig. 8(a) and (b).

These curves provide a baseline for performance comparisons, emphasizing the system’s responsiveness in steady-state environmental conditions.

#### Irradiance fluctuation and constant temperature

**Fig. 9:**
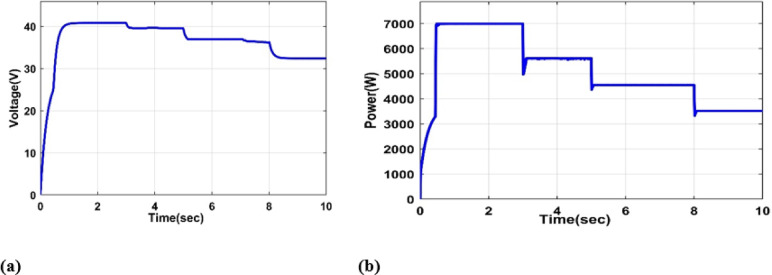
(**a**) PV output Voltage under variable irradiance and 25 °C, (**b**) PV output Power under variable irradiance and 25 °C.

In this part, we look at the PV system’s voltage and power outputs at different irradiance levels [1000 W/m2, 800 W/m2, 650 W/m2, 500 W/m2] and a temperature of 25 °C, as shown in Fig. 9(a, b).

It was observed from Fig. 9(a, b) that reduced radiation levels resulted in lower PV output power and voltage, which in turn caused a decline in the overall efficiency of the solar system.

#### Constant irradiance and variable temperature

**Fig. 10:**
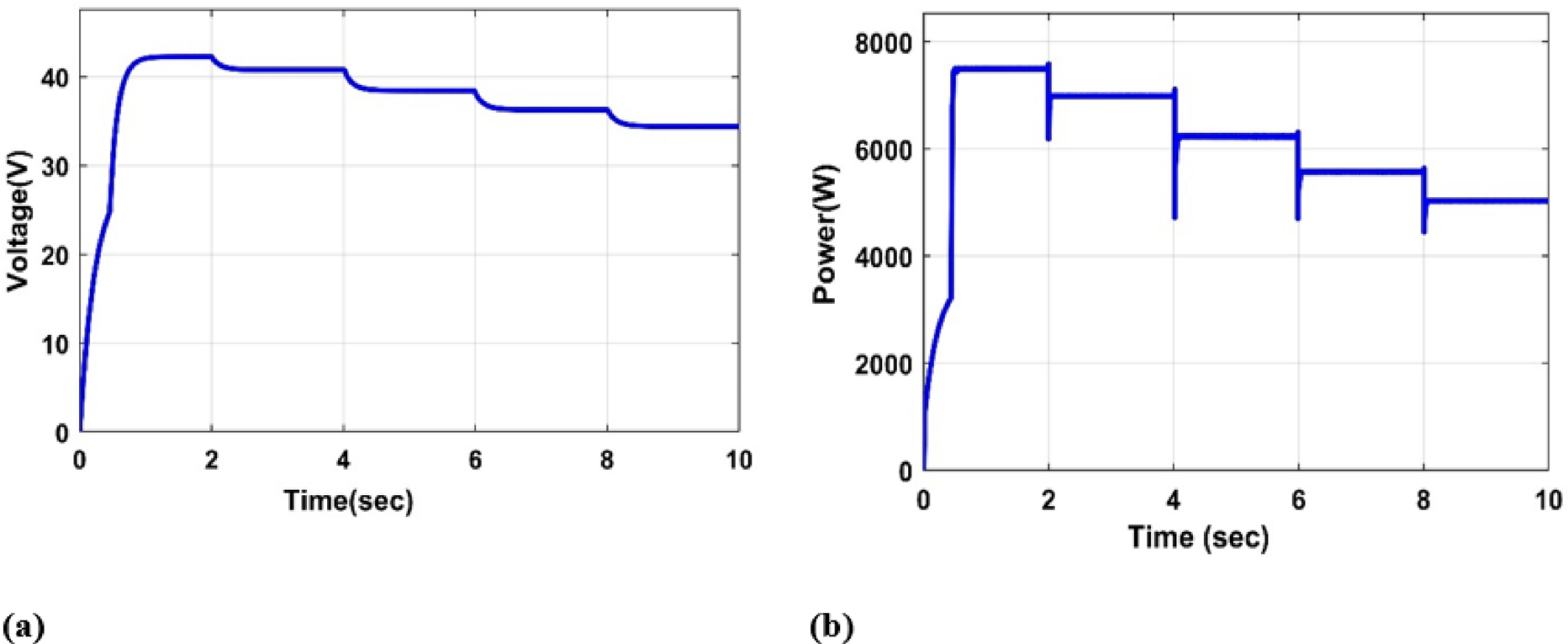
(**a**) PV output Voltage under constant irradiance and variable temperature (°C), (**b**) PV output Power under constant irradiance and variable temperature (°C).

In this section, we analyze the PV system’s voltage and power outputs at a fixed irradiation of 1000 W/m2 and a varied temperature of [5 °C, 25 °C, 55 °C, 80 °C, 100 °C] as shown in Fig. 10(a), (b).

From Fig. 10(a, b), it was observed that as the temperature increased, both the PV output power and voltage decreased, leading to a reduction in the solar system’s efficiency.

#### Variable irradiance and temperature

**Fig. 11:**
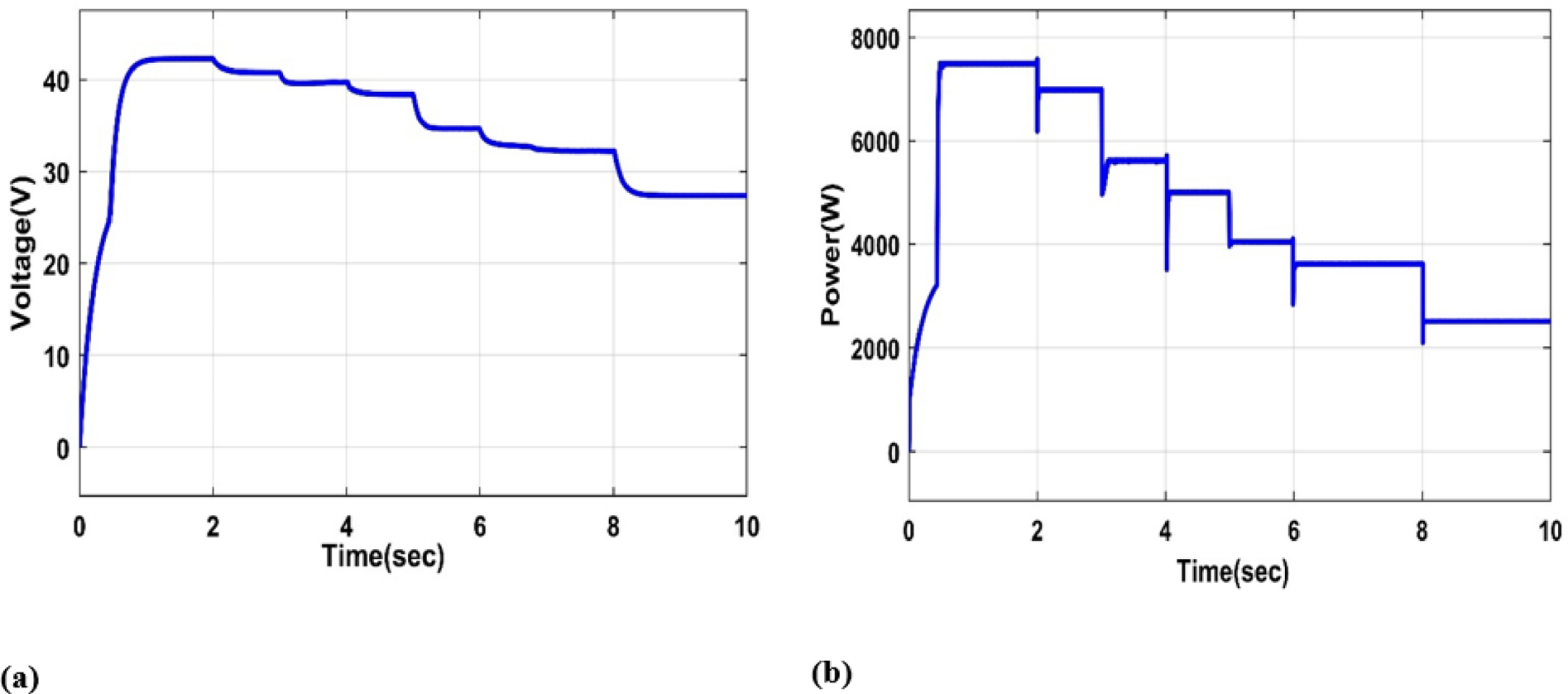
(**a**) PV output Voltage under variable irradiance(W/m2) and temperature (°C), (**b**) PV output Power under variable irradiance(W/m2) and temperature (°C).

In this part, we examine the voltage and power outputs of the PV system at a variable irradiation of [1000 W/m2, 800 W/m2, 650 W/m2, 500 W/m2] and a variable temperature of [5 °C, 25 °C, 55 °C, 80 °C, 100 °C], as shown in Fig. 11(a, b).

Figure 11(a, b) shows that when the two prior examples are combined, changing both the radiation and the temperature, the PV output power and output voltage fall, resulting in a decrease in the solar system’s efficiency.

### PEM electrolyzer results

#### Constant pressure and temperature

In this part, we analyzed the electrolyzer behavior under fixed pressure 1 atm and fixed temperature 25 °C and discovered that the quantity of hydrogen flow rate is equivalent to 22.32 L/min, and the electrolyzer efficiency is 67.45%, as shown in the following figures.

**Fig. 12:**
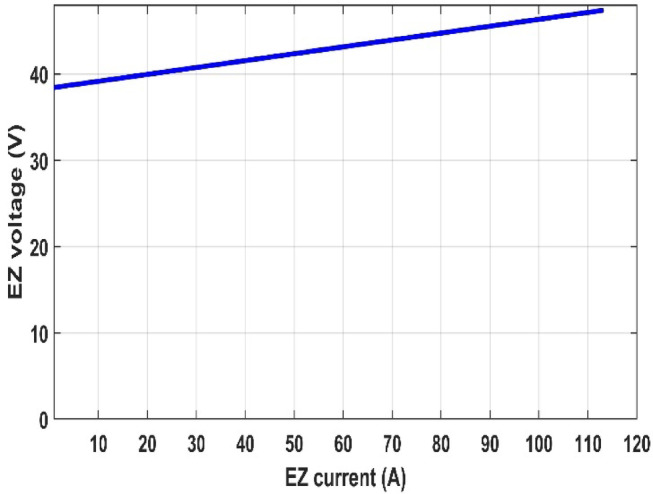
Polarization curve of PEM electrolyzer.

**Fig. 13:**
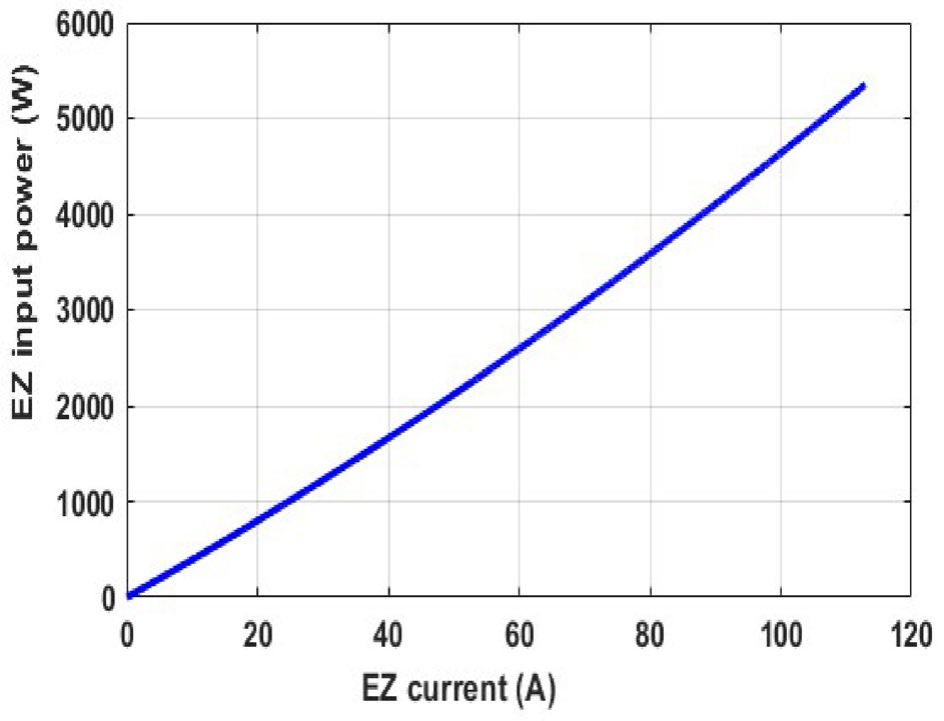
PEM electrolyzer input power versus current.

**Fig. 14:**
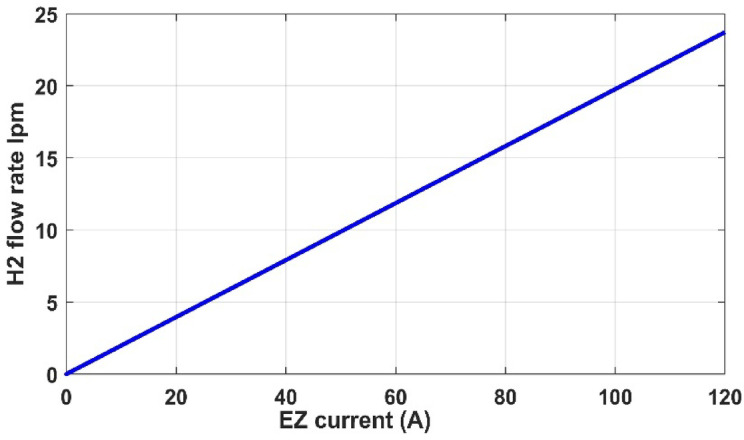
PEM electrolyzer hydrogen output flow rate versus current.

**Fig. 15:**
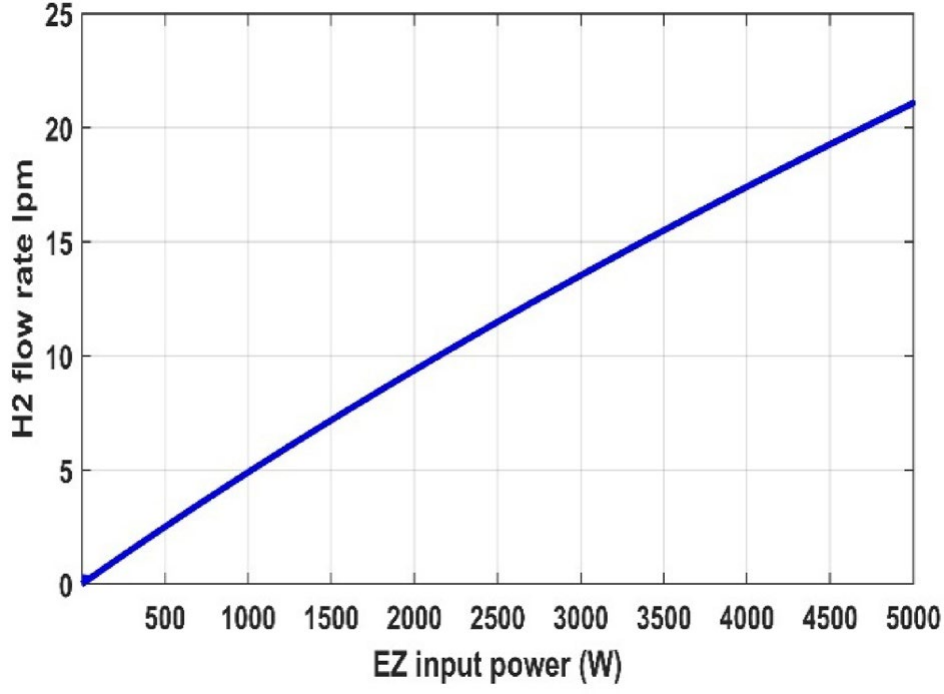
PEM electrolyzer hydrogen output flow rate versus input power.

**Fig. 16:**
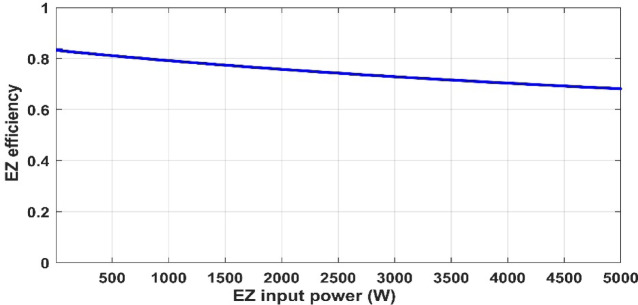
PEM electrolyzer efficiency versus input power.

**Fig. 17:**
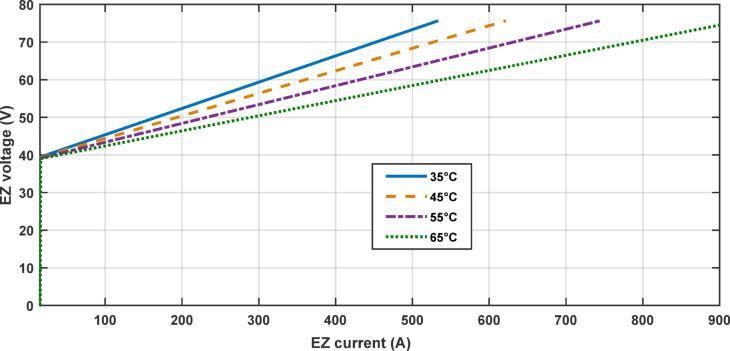
Polarization curve of PEMEZ (V-I) under variable temperature and constant pressure.

Figures 12, 13, 14, 15 and 16 clearly show a linear relationship between voltage and PEMEZ stack current under constant conditions. The slope of this curve can alter when the primary operational factors (pressure and temperature) are changed, as demonstrated in the next three sections. Figure 13 depicts the semi-linear relationship between the electrical power supplied to the PEMEZ and the current going across its membrane. Figures 14 and 15 show the semi-linear relationship between hydrogen generation rate and PEMEZ input power. Figure 16 depicts the fluctuation of the PEMEZ stack efficiency with the input power, as efficiency decreases with increasing power due to rising losses against the higher passing current across the membrane (Fig. 17).

#### PEMEZ results under variable temperature and constant pressure

In this part, we will analyze the behavior of PEMEZ fed from a solar system with a buck converter under variable temperature [35, 55, 65 °C] and a constant pressure equal to 1 atm, as shown in Fig. 12.

The amount of hydrogen created in each of the four preceding cases was analyzed and compared, as indicated in the (Table 3).

**Table 3:** The quantity of hydrogen produced under varying temperature conditions.

Temperature (°C)	Pressure (atm)	H2 flow rate (L/min)	Electrolyser current (A)
35	1 atm	109	533.5
45		131.1	621.6
55		162	744.7
65		208.2	928.5

This portion illustrates how an increase in temperature generates a decrease in the slope of the polarization curve and shows that when the temperature increases, the amount of hydrogen flow rate increases.

### Comparative analysis

In this section, the solar system’s findings and how the P&O MPPT technique was regulated using the FLC and PI, FOPI controllers were investigated. As indicated below, various algorithms were utilized to optimize PI and FOPI parameters.

#### PI controller behavior with three optimizations

In this part, the three methods were tested to ensure optimal tuning for the PI controller parameter. To provide a fair comparison of these algorithms, as shown in Tables 4 and 5, we ran all of them with the same population size, number of iterations, and boundary conditions. DCSO branch marking results when combined with other techniques.

**Table 4:** Comparative analysis of optimization algorithms for PI controller tuning and performance parameters.

Algorithms	Optimization settings/tuning				
	Max iteration	Population size	Upper boundary	Lower boundary	Algorithm parameter
DCSO	**50**	**25**	**[0.001**,**0.1]**	**[0**,**0]**	1- Golden Ratio (0.618) 2- Probability Constant (0.5)
PSO					1- Inertia Weight) 0.7( 2- Acceleration Coefficients(2)
GWO					1- Convergence factor Linear decrease from 2 to 0

**Fig. 18:**
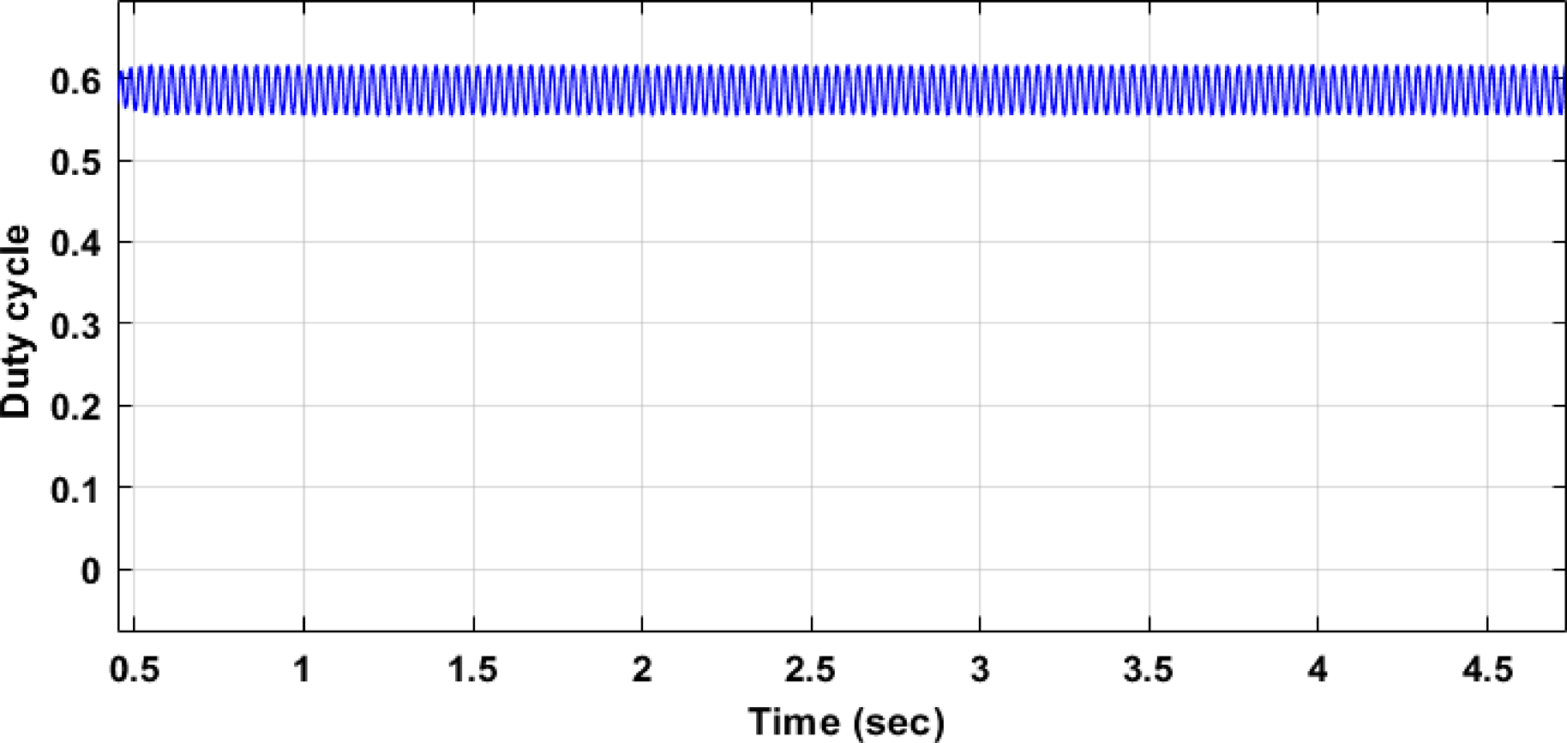
Duty cycle performance using PI controller tuned by DCSO.

**Fig. 19:**
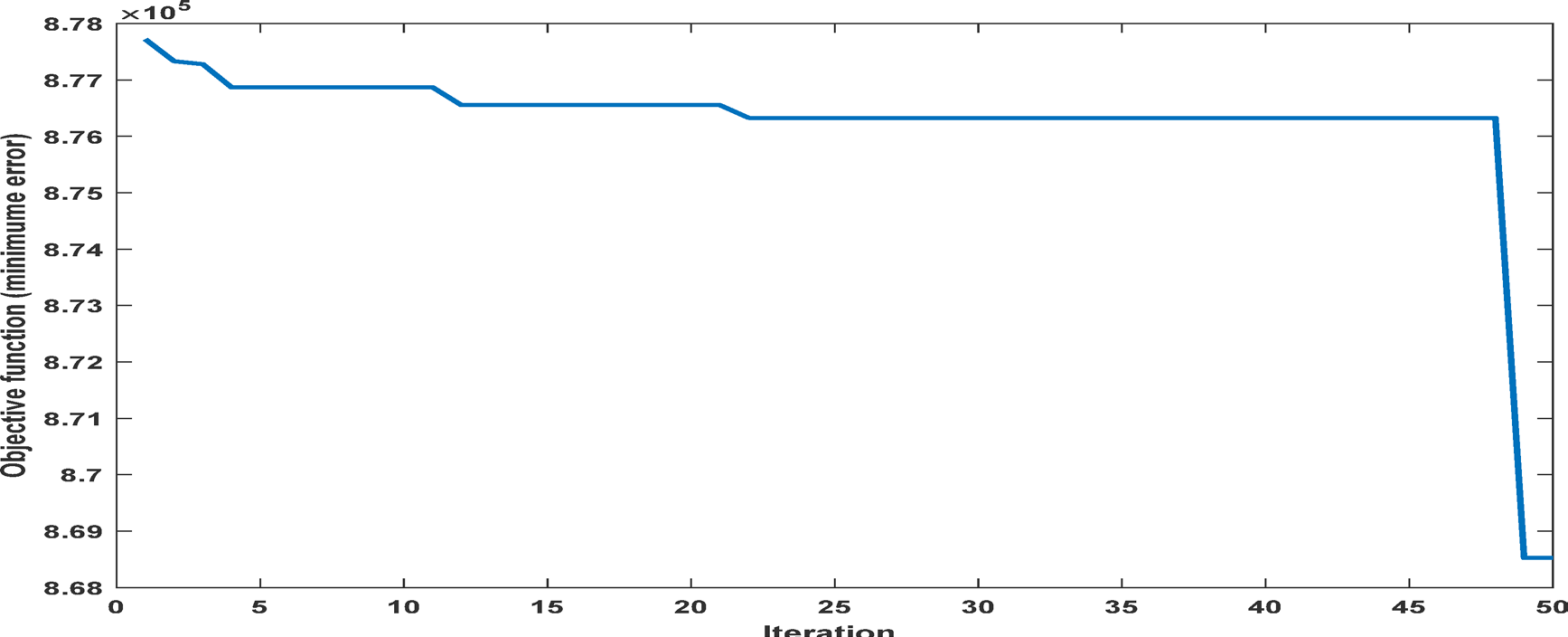
The convergence curve for PI tuning parameters using DCSO.

**Fig. 20:**
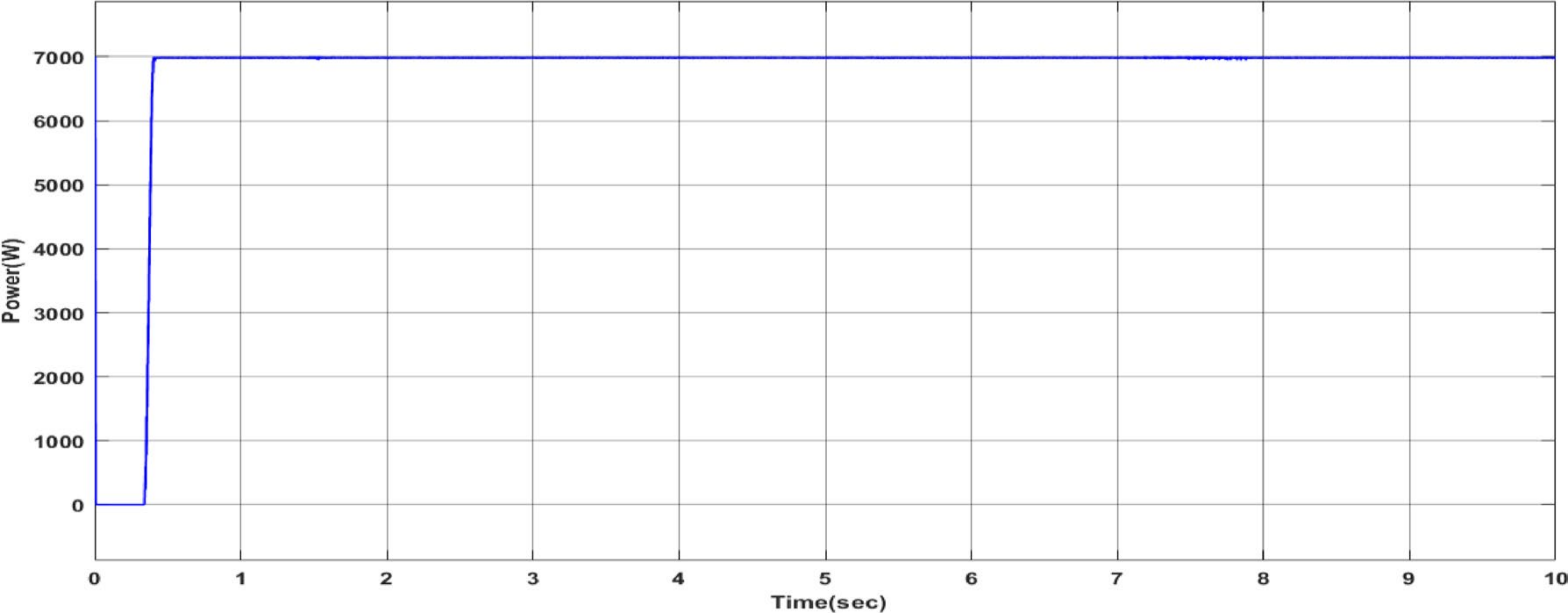
Power output of the PV cell with DCSO.

**Fig. 21:**
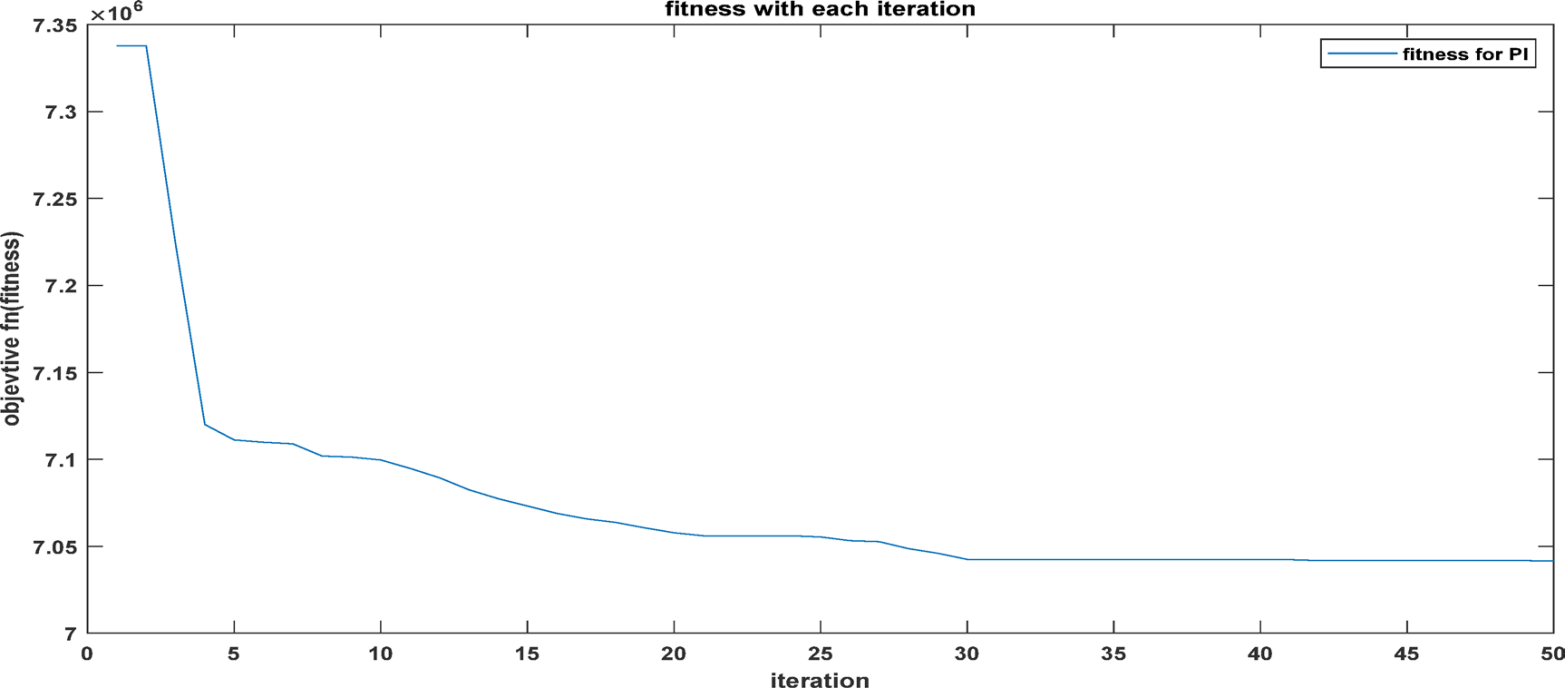
Convergence curve for PI tuning parameters using PSO.

**Fig. 22:**
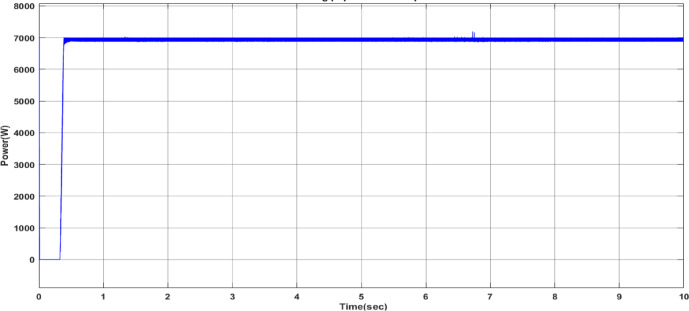
Power output of PV cell with PSO.

**Fig. 23:**
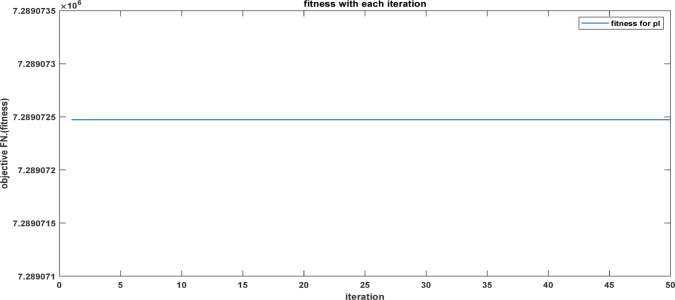
The convergence curve for PI tuning parameters using GWO.

**Fig. 24:**
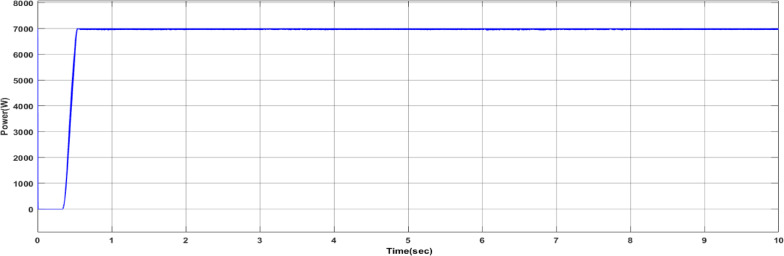
Power output of the PV cell with GWO.

**Table 5:** DCSO result for tuning PI controller parameters in comparison with other optimizers.

Algorithms	PV output power(W)	Output voltage (V)	PI controller parameters		Minimum fitness	Settling time (sec)	Ripple
			KP	KI			
DCSO	6987 W	45.14 V	0.0023	0.0967	8.6903e + 06	0.432	6.028e + 03
PSO	6922 W	44.78v	0.0141	0.0889	7.0532e + 06	0.36	6.030e + 03
GWO	6879	41.73	0.0009	0.0790	7.2891e + 06	0.504	6.016e + 03

From a close look at previous,, Figs. 18, 19, 20, 21, 22, 23 and 24; Table 5, it is clear that the best obtained results were for DCSO, PSO, and GWO respectively based on the fitness scale, where the best fitness was configured as the minimum value of the summation of square error between the actual output voltage from PV and reference voltage from P&O MPPT, Despite this, the optimal fitness values were near, and there was variability in terms of the time spent on the process of optimization.

#### FOPI controller behavior with three optimizations

This section evaluated the three methods to ensure optimal tuning of the FOPI controller parameters. To facilitate a fair comparison of these algorithms, as presented in Table 5, all procedures were executed with the same population size, number of iterations, and boundary conditions.

**Fig. 25:**
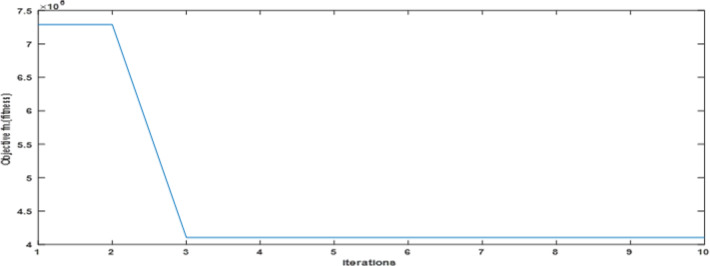
The convergence curve for FOPI tuning parameters using DCSO.

**Fig. 26:**
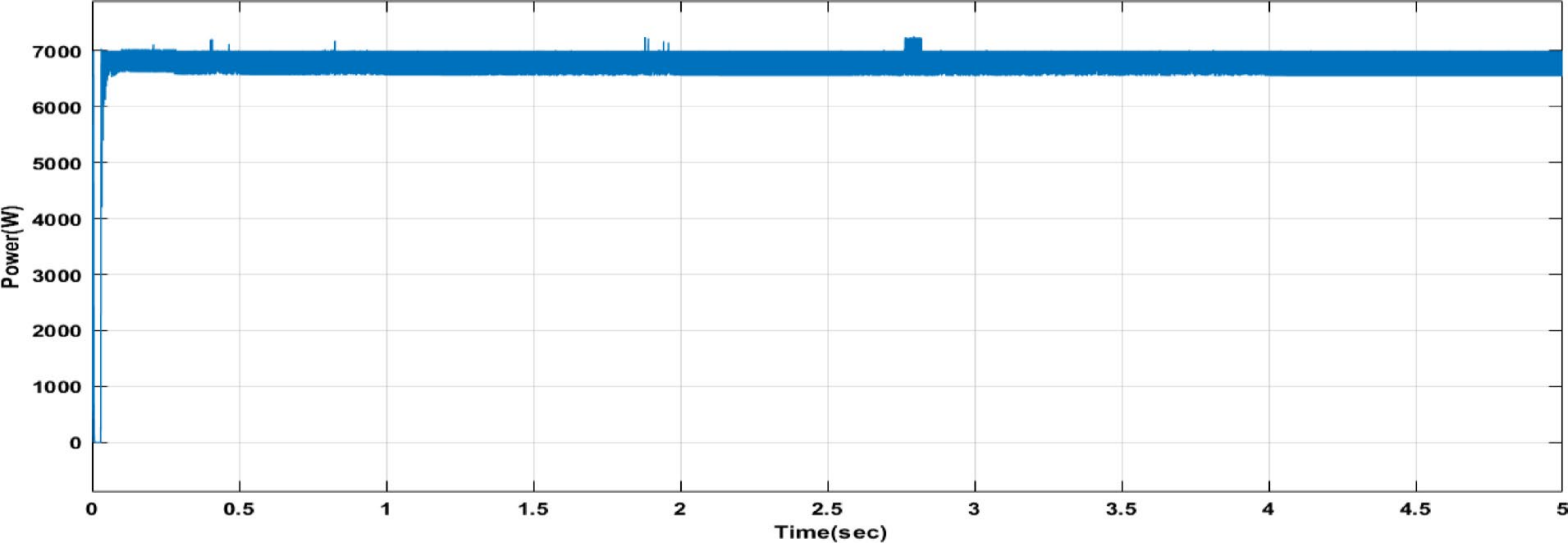
Power output of the PV system with DCSO.

**Fig. 27:**
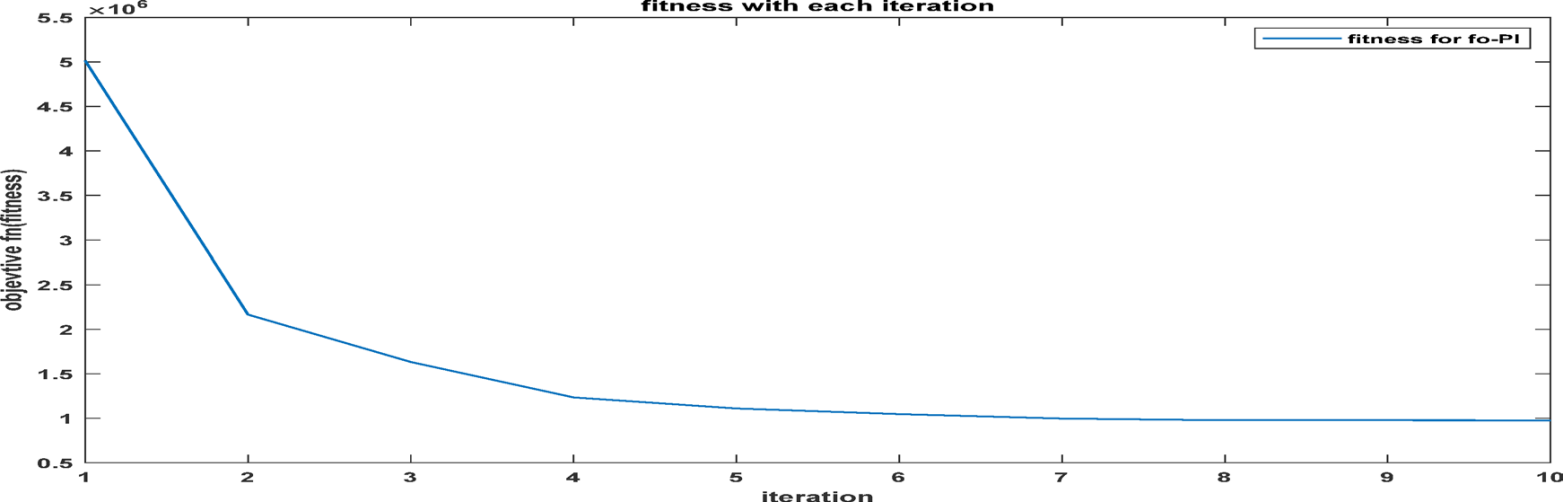
The convergence curve for FOPI tuning parameters using PSO.

**Fig. 28:**
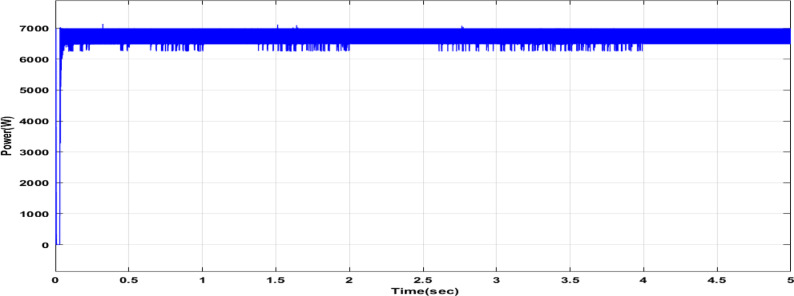
Power output of the PV system with PSO.

**Fig. 29:**
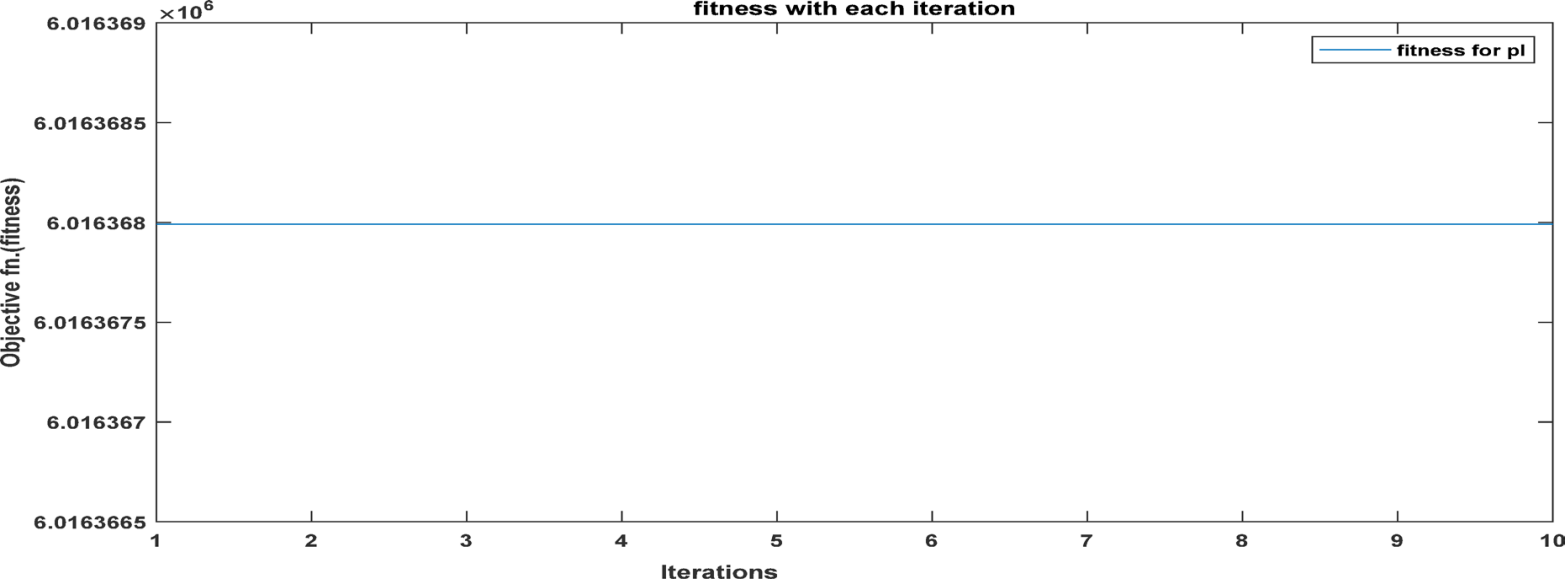
The convergence curve for FOPI tuning parameters using GWO.

**Fig. 30:**
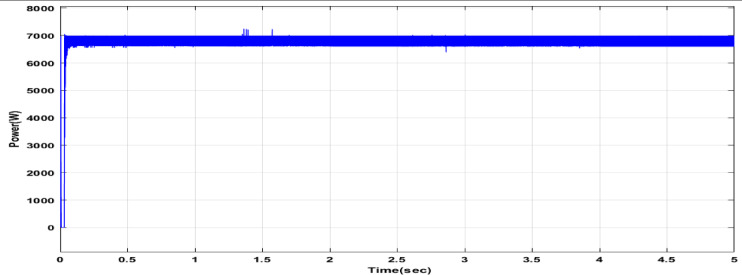
Power output of the PV system with GWO.

**Table 6:** DCSO result for tuning FOPI controller parameters in comparison with other optimizers.

Algorithms	PV output power(W)	Output voltage (V)	FOPI controller parameters			Minimum fitness	Settling time (sec)	Ripple
			K_*P*_	K_I_	λ			
DCSO	6767	44.52	0.0046	0.0084	0.0028	4.1039e06	0.144	6.073e + 03
PSO	6697	44.34	0.0045	0.0085	0.0062	1.6943e06	0.1512	6.223e + 03
GWO	6672	44.42	0.0048	0.0084	0.0080	6.0164e06	0.1589	6.176e + 03

It’s clear from the previous Figs. 25, 26, 27, 28, 29 and 30; Table 6 that the DCSO method produced results that were comparable when combined with other techniques. A closer examination of the table reveals that the best results, based on the fitness scale, were achieved by DCSO, PSO, and GWO, respectively. The fitness was defined as the minimum value of the summation of squared errors between the actual output voltage from the PV system and the reference voltage from the P&O MPPT. Although the optimal fitness values were similar, there were differences in the time required for the optimization process.

#### Three controllers: FLC, PIC, and FOPI behaviors

**Fig. 31:**
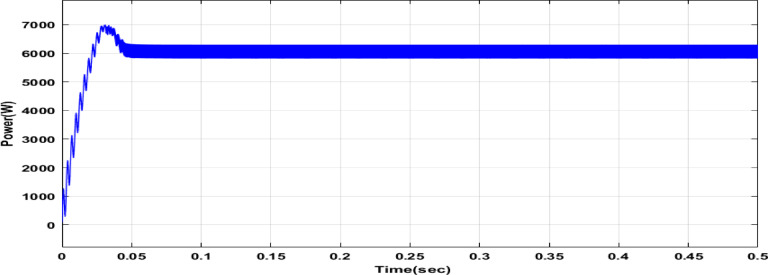
Power output of the PV system with FLC.

Table 7 The result of the fuzzy logic controller in comparison with other PI and FOPI controllers.

Figure 31 depicts the time-domain response of PV output power when the FLC is utilized. The major goal of this image is to show how the controller achieves its final working value. As illustrated, the FLC successfully drives the system to a power output of 6296 W. Although the response is smooth and free of huge, unexpected leaps, it takes longer to achieve the final value than DCSO-tuned controllers. This figure provides visible confirmation of the FLC’s ability in maintaining a continuous power supply to the PEM electrolyzer, despite its reduced efficiency in extracting the maximum possible power.

**Table 7:** Comparison between PI-DCSO, FOPI-DCSO, and FLC.

Controller	PV output power(W)	Output voltage(V) on the buck converter terminals	Settling time (sec)	Ripple
PI-DCSO	6987	45.14	0.432	6.028e + 03
FOPI-DCSO	6767	44.52	0.144	6.073e + 03
FLC	6296	41.73	0.216	5.998e + 03

A comparison was made between the Fuzzy Logic Controller (FLC), the PI controller optimized using several algorithms (GWO, PSO, and DCSO), and the Fractional-Order PI (FOPI) controller utilizing the same optimization suite. As shown in Fig. 31, the FLC’s tracking capabilities was significantly limited, as it failed to accurately achieve or hold the Maximum Power Point (MPP) in the evaluated conditions. This performance disparity is related to the FLC’s fixed membership functions, which may not adjust quickly to fast irradiance changes. In contrast, the optimized PI and FOPI controllers displayed improved tracking accuracy, effectively attaining the MPP with low error, which validates the use of metaheuristic algorithms for controller tuning in PV systems. MPPT control is a technique for tracking the maximum power point under the impact of radiation 1000 W/m2 and temperature 25°C while feeding a PEM electrolyzer. Based on the results in Table 7, a thorough comparison of the three controllers indicates unique performance trade-offs. The PI-DCSO controller obtained the greatest peak power extraction of 6987 W, successfully maximizing the PV system’s capacity. This supremacy in power tracking is due to differential creative search Optimization (DCSO), which precisely optimized the Kp and K_i_ gains to match the operating point with the MPP. However, in terms of temporal reaction, the FOPI-DCSO had the quickest settling time (0.144 s), which was much faster than the normal PI-DCSO (0.432 s).The fractional-order operators (λ and µ) give more degrees of freedom, allowing the controller to efficiently suppress transients, resulting in this improvement. On the other hand, the FLC delivered a balanced performance but struggled to match the DCSO-tuned controllers’ steady-state accuracy, resulting in the lowest power output (6296 W). The high accuracy reported here, particularly with the PI-DCSO, outperforms the findings in Ref.^4^, confirming that combining advanced metaheuristic optimization with robust modeling significantly reduces the ‘chattering’ effect and improves the overall efficiency of the PV-PEM hydrogen production system. In further work, we propose to examine the performance of P&O MPPT with adaptive FLC and a hybrid fuzzy _PI controller. The current results indicate higher accuracy due to the utilization of advanced modeling approaches in comparison to the results presented in the Ref.^4^.

#### Comparison of three DC/DC converters

This section compares the three types of DC-DC converters: buck, boost, and buck-boost converters, have component values as shown in Table 8, in terms of studying their effect on PV output power and PV efficiency, as well as their effect on electrolyzer efficiency and the amount of produced hydrogen. Every converter topology’s steady-state analysis determines the design of the reactive components (L and C). The main design parameters are the inductor current ripple (∆I_L_) and the output voltage ripple (∆V_out_)^39^.

**Table 8:** DC/DC power converters’ component values.

Components	Buck converter	Boost converter	Buck-boost converter
Inductance (H)	$\:\frac{(1-D)\varDelta\:\mathrm{V}\mathrm{o}\mathrm{u}\mathrm{t}}{{f}_{s\:}\:{\varDelta\:I}_{L\:}}$ 1.47e-5	$\:\frac{D{V}_{in}}{{f}_{s\:}\:{\varDelta\:I}_{L\:}}$ 0.0000125	$\:\frac{D{V}_{in}}{{f}_{s\:}\:{\varDelta\:I}_{L\:}}$ 0.000036
Capacitance(F)	$\:\frac{{\varDelta\:I}_{L\:}}{{8f}_{s\:}{\varDelta\:V}_{out\:}}$ 0.07592	$\:\frac{{I}_{out}D}{{f}_{s\:}{\varDelta\:V}_{out\:}}$ 0.002	$\:\frac{{I}_{out}D}{{f}_{s\:}{\varDelta\:V}_{out\:}}$ 0.008
Frequency (KHZ)	5	5	5

Determine the best proportional-integral (K_p_, K_i_) gains for minimizing the system error. By reducing the selected fitness function (Integral Absolute Error - IAE), the improved PI controller may dynamically modify the duty cycle (D) to guarantee a rapid and steady response, maintaining the output voltage at the intended setpoint despite variations in solar irradiation.

**Fig. 32:**
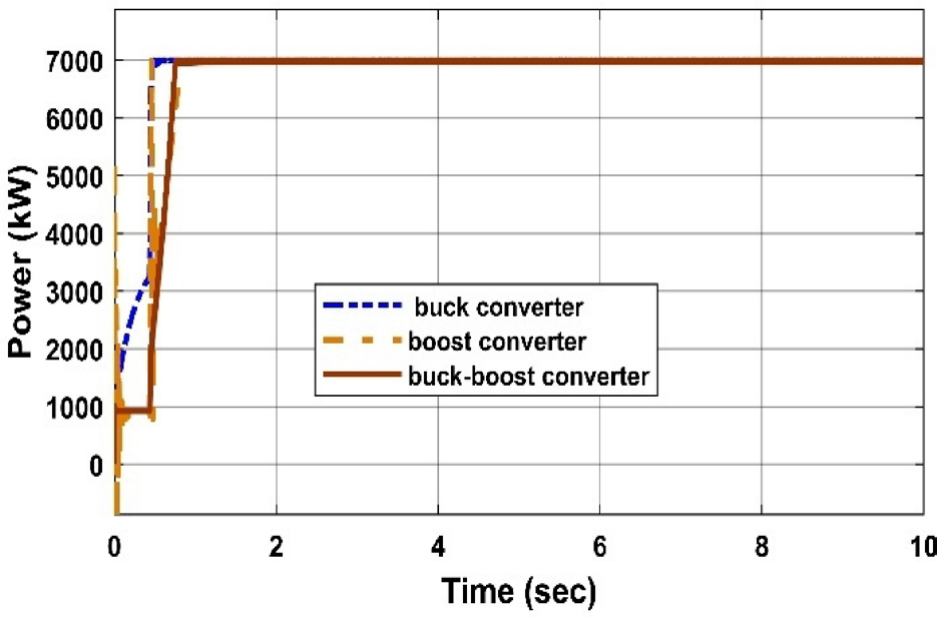
PV output Power under 1000 W/m^2^ and 25 °C using different converters.

**Fig. 33:**
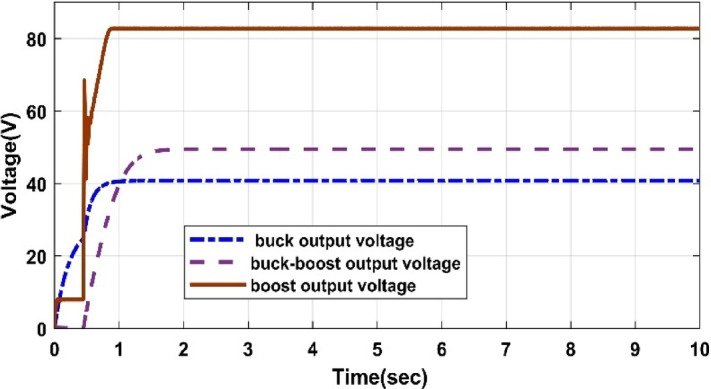
Different converter output voltages under 1000 W/m^2^ and 25 °C.

**Fig. 34:**
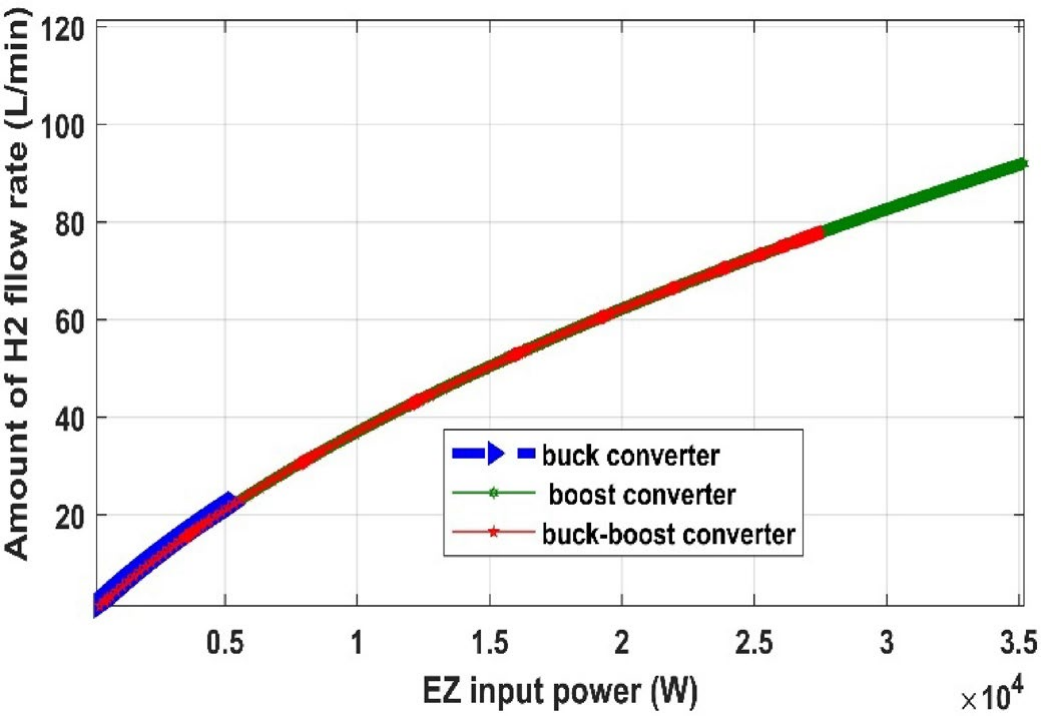
PEM electrolyzer hydrogen flow rate versus input power with different converters.

**Fig. 35:**
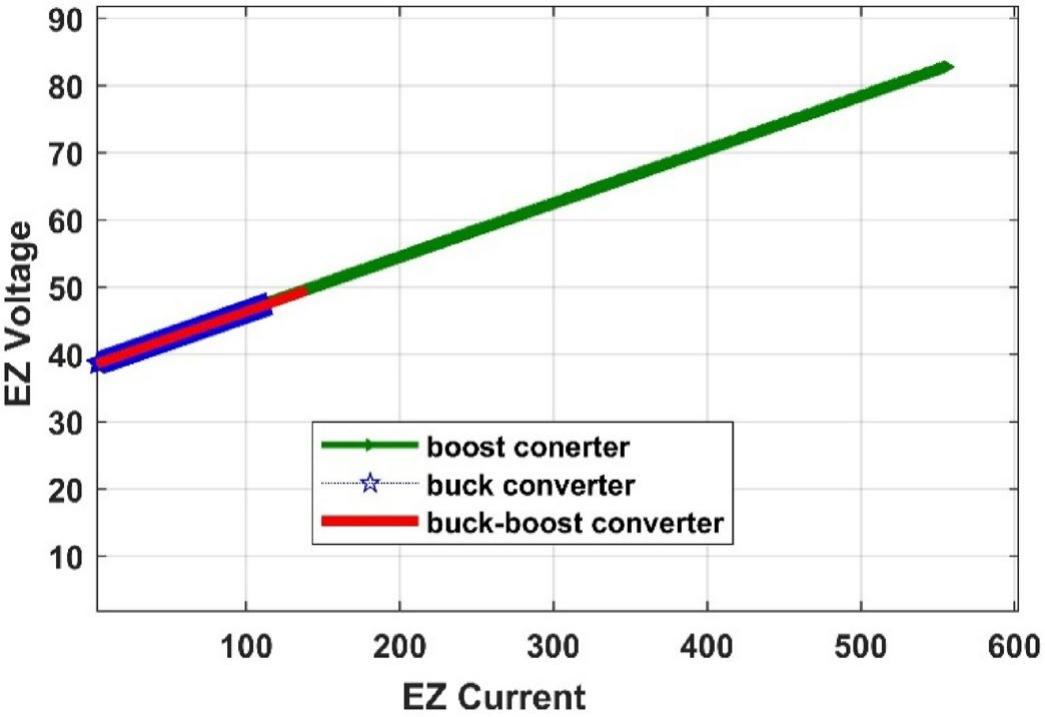
PEM electrolyzer polarization curve under different converters.

**Table 9:** Comparison of several DC-DC converters.

Converter types	PV output power (W)	Converter output voltage (V)	PV system efficiency (%)	Amount of H2 produced by PEMEZ(L/min)	EZ efficiency (%)
Buck converter	6994	41.39	34.97	16.74	70.48
Boost converter	6992	82.74	34.96	109.9	38.6
Buck-Boost converter	6988	49.5	34.94	27.56	64.57

The comparative study shown in Figs. 32, 33, 34 and 35, as well as Table 9, demonstrates a key trade-off between hydrogen generation rate and PEM electrolyzer efficiency. While the Boost converter produced the maximum hydrogen flow rate (109.9 L/min), it did so at a considerable loss of electrolyzer efficiency (38.6%), most likely because to the high voltage stress, which increases internal ohmic losses. In contrast, the Buck converter provided the best performance for this integrated system, maintaining the maximum electrolyzer efficiency of 70.48% and a PV system efficiency of 34.97%. This suggests that the Buck topology offers a better impedance match between the PV source and the PEM stack. As a result, the Buck converter is recommended as the best interface for sustainable hydrogen generation, establishing a balance between energy harvesting and the lifespan of the electrolysis unit.

### System performance limitations

The examination of Table 9 reveals that the PEM electrolyzer is the primary component limiting overall system performance. The PV array and DCSO-controller have good energy harvesting efficiency (~ 35% and 99.8% tracking, respectively), however the electrolyzer’s efficiency reduces to 38.6% with faulty voltage matching (Boost scenario). This demonstrates that the interface matching between the converter and the electrolyzer is the main barrier for hydrogen generation efficiency.

## Conclusion

This study optimized green hydrogen production using a PV-powered PEM electrolyzer by comparing MPPT control strategies. The PI-DCSO controller achieved the highest PV output power (6987 W) and fastest settling time (0.432 s), outperforming FOPI-DCSO (6767 W) and FLC (6296 W). The PEM electrolyzer produced 22.32 L/min of hydrogen at 67.45% efficiency under standard conditions (1 atm, 25 °C), with performance improving at higher temperatures (208.2 L/min at 65 °C) but declining at elevated pressures (63.07 L/min at 30 atm). The buck converter proved most efficient (34.97%) for PV-to-electrolyzer power transfer. These results demonstrate that metaheuristic-optimized PI control maximizes renewable hydrogen production, offering a viable path toward sustainable energy solutions. The findings highlight the critical role of controller selection and operating conditions in system performance. While PI-DCSO delivered optimal power tracking, temperature and pressure significantly influenced hydrogen yield, with trade-offs between efficiency and production rates. The thermal behavior of the PEM electrolyzer conforms with electrochemical theory. However, the inclusion of a sophisticated DCSO-based control guarantees that the system performs at its optimal electrical efficiency, increasing hydrogen production per watt generated.Actionable ideas for scaling up green hydrogen technology to address energy and environmental concerns. As part of future work, the scope of this research will be expanded to include a comparison examination of the proposed DCSO and other fairly recent metaheuristic techniques (2023–2025) to further analyze efficiency and settling times. Furthermore, a practical execution utilizing Hardware-in-the-Loop (HIL) or an experimental prototype will be performed to assess the practicality and real-world applicability of the presented scenarios. This step will evaluate the simulation findings while identifying any operational or technical issues that may arise during actual deployment, such as the requirement for hybrid controllers or adaptive tuning in highly dynamic situations.additionally to provide a more multi-dimensional assessment of the proposed control strategies, future research will focus on expanding the performance evaluation framework. This will involve the integration of additional error performance indices, such as **integral square error (ISE)** and **mean square error (MSE)**, alongside a comprehensive statistical analysis. Such an expansion will offer deeper insights into the transient and steady-state precision of the DCSO-tuned controllers across a wider range of dynamic operating conditions.

## Data Availability

Upon reasonable request, the corresponding author will provide the research data generated and analyzed during this study, including simulation models applied using MATLAB/SIMULINK version: R2023a, as well as the resulting figures and comparison tables related to solar and hydrogen energy systems, for verification or reuse in future studies.

## References

[1] Hassan, Q., Tabar, V. S., Sameen, A. Z., Salman, H. M. & Jaszczur, M. A review of green hydrogen production based on solar energy; techniques and methods. *Energy Harvesting Syst.* **11** (1), 20220134 (2024).

[2] Awad, M. et al. A review of water electrolysis for green hydrogen generation considering PV/wind/hybrid/hydropower/geothermal/tidal and wave/biogas energy systems, economic analysis, and its application. *Alexandria Eng. J.* **87**, 213–239. 10.1016/j.aej.2023.12.032 (2024).

[3] Ikuerowo, T., Bade, S. O., Akinmoladun, A. & Oni, B. A. The integration of wind and solar power to water electrolyzer for green hydrogen production. *Int. J. Hydrogen Energy*. **76**, 75–96 (2024).

[4] Awad, M. et al. Design and analysis of photovoltaic/wind operations at MPPT for hydrogen production using a PEM electrolyzer: Towards innovations in green technology. *PLoS One*. **18** (7). 10.1371/journal.pone.0287772 (2023). 10.1371/journal.pone.0287772 PMC10358930 37471326

[5] Çakmak, F., Aydoğmuş, Z. & Tür, M. R. Analysis of open circuit voltage MPPT method with analytical analysis with perturb and observe (P&O) MPPT method in PV systems. *Electr. Power Compon. Syst.* **52** (9), 1528–1542 (2024).

[6] Hassan, Q., Sameen, A. Z., Salman, H. M. & Jaszczur, M. Large-scale green hydrogen production via alkaline water electrolysis using solar and wind energy. *Int. J. Hydrogen Energy*. **48** (88). 10.1016/j.ijhydene.2023.05.126 (2023).

[7] IRENA, Green Hydrogen Cost Reduction. (2020).

[8] Grimm, A., de Jong, W. A. & Kramer, G. J. Renewable hydrogen production: A techno-economic comparison of photoelectrochemical cells and photovoltaic-electrolysis. *Int. J. Hydrogen Energy*. **45** (43). 10.1016/j.ijhydene.2020.06.092 (2020).

[9] Marius Holst, S., Aschbrenner, T., Smolinka, C., Voglstätter, G. & Grimm Cost Forecast for Low-Temperature Electrolysis - Technology driven bottom-up prognosis for PEM and Alkaline water electrolysis systems, (2021).

[10] Laib, A., Krim, F., Talbi, B., Kihal, A. & Feroura, H. Improved control of three phase dual-stage grid-connected PV system based on a predictive control strategy. (2018).

[11] Kihel, A., Krim, F. & Laib, A. MPPT voltage oriented loop based on integral sliding mode control applied to the boost converter. In *2017 6th International Conference on Systems and Control (ICSC)*, pp. 205–209. 10.1109/ICoSC.2017.7958687 (2017).

[12] Kanouni, B. et al. High-accurate parameter identification of PEMFC using advanced multi-trial vector-based sine cosine meta-Heuristic algorithm. *IEEE Access.* **13**, 170827–170843. 10.1109/ACCESS.2025.3614048 (2025).

[13] Kanouni, B., Badoud, A. E. & Mekhilef, S. A SMC-based MPPT controller for proton exchange membrane fuel cell system. In *2022 19th International Multi-Conference on Systems, Signals & Devices (SSD)*, pp. 527–531. 10.1109/SSD54932.2022.9955704 (2022).

[14] Grimm, A., de Jong, W. A. & Kramer, G. J. Renewable hydrogen production: A techno-economic comparison of photoelectrochemical cells and photovoltaic-electrolysis. *Int. J. Hydrogen Energy*. **45** (43), 22545–22555 (2020).

[15] Olivier, P., Bourasseau, C. & Bouamama, P. B. Low-temperature electrolysis system modelling: A review. *Renew. Sustain. Energy Rev.* **78**, 280–300 (2017).

[16] Li, G. et al. Optimization of hydrogen production system performance using photovoltaic/energies (Basel), **17**, 21 10.3390/en17215405. (2024).

[17] Brezak, D., Kovač, A. & Firak, M. MATLAB/Simulink simulation of low-pressure PEM electrolyzer stack. *Int. J. Hydrogen Energy*. **48** (16), 6158–6173. 10.1016/j.ijhydene.2022.03.092 (2023).

[18] Mazumdar, D., Biswas, P. K., Sain, C. & Ustun, T. S. GAO optimized sliding mode based reconfigurable step size Pb&O MPPT controller with grid integrated EV charging station. *IEEE Access.* **12**, 10608–10620. 10.1109/ACCESS.2023.3344275 (2024).

[19] Mazumdar, D. et al. Optimizing MPPT control for enhanced efficiency in sustainable photovoltaic microgrids: A DSO-based approach. *Int. Trans. Electr. Energy Syst.* **2024**10.1155/2024/5525066 (2024).

[20] Mazumdar, D., Biswas, P. K., Sain, C., Ahmad, F. & Al-Fagih, L. Developing a resilient framework for electric vehicle charging stations harnessing solar energy, standby batteries and grid integration with advanced control mechanisms. *Energy Sci. Eng.* **12** (10), 4355–4370. 10.1002/ese3.1888 (2024).

[21] Albarghot, M., Sasi, M. & Rolland, L. Modeling and experimental results of a PEM electrolyzer powered by a solar panel. *J. Energy Power Eng.* **10** (12). 10.17265/1934-8975/2016.12.009 (2016).

[22] Mazumdar, D., Biswas, P. K., Sain, C., Ahmad, F. & Al-Fagih, L. An enhanced MPPT approach based on CUSA for grid-integrated hybrid electric vehicle charging station. *Int. J. Energy Res.* **2024**, 10.1155/2024/7095461 (2024).

[23] Baharudin, N. H., Mansur, T. M. N. T., Hamid, F. A., Ali, R. & Misrun, M. I. Topologies of DC-DC converter in solar PV applications. *Indonesian J. Electr. Eng. Comput. Sci.* **8** (2). 10.11591/ijeecs.v8.i2.pp368-374 (2017).

[24] Mishra, D. P., Senapati, R. & Salkuti, S. R. Comparison of DC-DC converters for solar power conversion system. *Indonesian J. Electr. Eng. Comput. Sci.* **26** (2). 10.11591/ijeecs.v26.i2.pp648-655 (2022).

[25] Eseosa, O. & Kingsley, I. Comparative study of MPPT techniques for photovoltaic systems. *Saudi J. Eng. Technol.* **05** (02). 10.36348/sjet.2020.v05i02.002 (2020).

[26] Zand, S. J., Hsia, K. H., Eskandarian, N. & Mobayen, S. Improvement of self-predictive incremental conductance algorithm with the ability to detect dynamic conditions. *Energies (Basel)*. **14** (5). 10.3390/en14051234 (2021).

[27] Banakhr, F. A. & Mosaad, M. I. High performance adaptive maximum power point tracking technique for off-grid photovoltaic systems. *Sci. Rep.* **11** (1). 10.1038/s41598-021-99949-8 (2021). 10.1038/s41598-021-99949-8 PMC8516987 34650159

[28] Kumar, A. & Kumar, V. Hybridized ABC-GA optimized fractional order fuzzy pre-compensated FOPID control design for 2-DOF robot manipulator. *AEU - Int. J. Electron. Commun.* **79**10.1016/j.aeue.2017.06.008 (2017).

[29] Chiu, C. S. T-S fuzzy maximum power point tracking control of solar power generation systems. *IEEE Trans. Energy Convers.* **25** (4). 10.1109/TEC.2010.2041551 (2010).

[30] Hüner, B. Mathematical modeling of an integrated photovoltaic-assisted PEM water electrolyzer system for hydrogen production. *Int. J. Hydrogen Energy*. **79**, 594–608 (2024).

[31] Göbek, A. & Yurtcan, A. B. *Polymer Electrolyte Membrane Fuel Cell (PEMFC) Membranes, in Prospects of Hydrogen Fueled Power Generation* pp. 17–51 (River, 2024).

[32] Araújo, H. F., Gómez, J. A. & Santos, D. M. F. Proton-exchange membrane electrolysis for green hydrogen production: Fundamentals, cost breakdown, and strategies to minimize platinum-group metal content in hydrogen evolution reaction electrocatalysts. *Catalysts* **14** (12), 845 (2024).

[33] Kaur, G. & Cells, P. E. M. F. Fundamentals, advanced technologies, and practical application. 10.1016/C2020-0-00143-X (2021).

[34] Marefatjouikilevaee, H., Auger, F. & Olivier, J. C. Static and dynamic electrical models of proton exchange membrane electrolysers: A comprehensive review. *Energies (Basel)*. **16** (18). 10.3390/en16186503 (2023).

[35] Ding, D. et al. Review of electrolyser modeling in wind power and photovoltaic electrolysis for hydrogen production. In *2023 IEEE 2nd International Power Electronics and Application Symposium (PEAS)*, pp. 1043–1046. 10.1109/PEAS58692.2023.10395346 (2023).

[36] Folgado, F. J., González, I. & Calderón, A. J. Simulation platform for the assessment of PEM electrolyzer models oriented to implement digital Replicas. *Energy Convers. Manag*. **267**, 115917 (2022).

[37] Anastasiadis, A. G., Papadimitriou, P., Vlachou, P. & Vokas, G. A. Management of hybrid wind and photovoltaic system electrolyzer for green hydrogen production and storage in the presence of a small fleet of hydrogen vehicles—An economic assessment. *Energies (Basel)*. **16** (24). 10.3390/en16247990 (2023).

[38] Duankhan, P., Sunat, K., Chiewchanwattana, S. & Nasa-ngium, P. The differentiated creative search (DCS): Leveraging differentiated knowledge-acquisition and creative realism to address complex optimization problems. *Expert Syst. Appl.* **252**, 123734. 10.1016/j.eswa.2024.123734 (2024).

[39] Paul, M. Design and analysis of DC-DC converters for photovoltaic systems. *Asian J. Electr. Sci.* **8**, 1–4. 10.51983/ajes-2019.8.S1.2318 (2019).

